# Effects of Different Sources of *Armillaria mellea* Co-Cultivation on the Quality and Soil Microecology of *Gastrodia elata*

**DOI:** 10.3390/plants15091329

**Published:** 2026-04-27

**Authors:** Li Dong, Chengcui Yang, Xinting Su, Duo Han, Dongsu Zhao, Zhongyan Tang, Fen Xiong, Yinzhu Dong, Xiaodan Wang, Yonghong He, Shunqiang Yang

**Affiliations:** 1 College of Plant Protection, Yunnan Agricultural University, Kunming 650201, China; 14769158780@163.com (L.D.); sxtsyw@163.com (X.S.); wxd18787100724@163.com (X.W.); 2 Yunnan Key Laboratory of Gastrodia Elata and Fungal Symbiotic Biology, Zhaotong 657000, China; ycjgxcm@163.com (C.Y.); 18887212025@163.com (D.H.); 202215060110@stu.ztu.edu.cn (D.Z.); 13988395247@nwsuaf.edu.cn (Z.T.); 202215060131@stu.ztu.edu.cn (F.X.); 202215060143@stu.ztu.edu.cn (Y.D.); 3 Yunnan Yiliang Courtyard of Gastrodia Elata Science and Technology, Zhaotong 657000, China; 4 College of Agriculture and Life Sciences, Zhaotong University, Zhaotong 657000, China; 5 Yunnan Key Laboratory of Smart Villages and Agri-Cultural-Tourism Integration, Zhaotong 657000, China

**Keywords:** *Gastrodia elata*, *Armillaria mellea*, gastrodins, parishins, soil microorganism

## Abstract

To investigate the effects of different *Armillaria mellea* (*A. mellea*) sources on *Gastrodia elata* (GE) yield, quality, and soil microecology, five *A. mellea* of difference sources (M1–M5) were co-cultivated with Zhaotong GE at two sites. M5 co-cultivation produced the highest yields (fresh weight) at both Xiaocaoba, Yiliang (XCB) and Wanchang, Zhenxiong (ZXWC), reaching 3239 g/m^2^ and 2550 g/m^2^. For quality, M1 resulted in the highest total gastrodin and parishin content at XCB, while M3 was superior at ZXWC. Post-cultivation, soil pH increased across all treatments. Soil nutrients increased with M1 and M2 at XCB, and with M4 at ZXWC. In GE rhizosphere soil samples, *Proteobacteria*, *Acidobacteria*, and *Actinobacteria* were dominant bacterial phyla, with *Proteobacteria* abundance decreasing. The dominant fungal phyla were *Ascomycota*, *Basidiomycota*, and *Mucoromycota*; *Basidiomycota* abundance increased in all soils, while *Ascomycota* increased only with M1 and M2. M3-treated soils had the highest Gram-negative bacteria abundance, and M1-treated soils were enriched with saprotrophic fungi. This study has several limitations. The conclusions were drawn indirectly with the host (GE) as the focus, and the *A. mellea* were not identified to clarify potential genetic differences. Future research should integrate fungal omics analyses for a more in-depth investigation.

## 1. Introduction

*Gastrodia elata* (GE), also known as Chijian, Guiduyou, Dingfengcao, and Limu, is a perennial herb belonging to the family Orchidaceae [1,2]. It is rich in bioactive constituents such as gastrodin (GAS), p-hydroxybenzyl alcohol (HBA), and parishins (E, B, C, and A), which are recognized as the primary bioactive components [3]. These compounds not only endow GE with high medicinal and edible value but also serve as critical criteria for evaluating the quality of this herb [4,5,6]. Among these, GAS and HBA are listed as crucial indicators for evaluating the quality of GE in the Chinese Pharmacopoeia.

Unlike other orchids, GE is an achlorophyllous plant lacking roots and leaves, and its growth and development depend entirely on symbiosis with *Armillaria mellea* (*A. mellea*). The quality of GE is determined by the robustness of this symbiotic relationship [7]. The expression changes in the antifungal protein gene *GAFP*, as well as the key genes for strigolactone biosynthesis and transport—carotenoid cleavage dioxygenases (CCDs) and ATP-binding cassette transporters (PDRs)—in GE, play a critical regulatory role in the establishment and maintenance of its symbiotic relationship with *A. mellea* [8]. Controlled experiments have demonstrated that strigolactones can promote the growth and hyphal branching of *A. mellea*, thereby facilitating the establishment of symbiosis between GE and *A. mellea*. Differences in growth characteristics, substrate decomposition rate, and enzyme activity among *A. mellea* strains lead to variations in nutrient supply to GE, thereby exerting significant impacts on the agronomic traits and contents of main bioactive components of GE. A substantial body of research has demonstrated that the concentrations of key bioactive constituents in GE, including GAS, HBA, and parishins (E, B, C, and A), are intricately associated with the quality of its symbiotic fungus, *A. mellea*. Screening suitable strains for co-cultivation of *A. mellea* and GE can improve the yield and GAS content of GE to a certain extent [9,10,11]. Therefore, investigating the effects of *A. mellea* from different sources on the growth and quality of GE is crucial for the future development of the GE industry.

Symbiotic cultivation of GE with *A. mellea* can alter the soil microecology. Studies have shown that cultivating GE on barren sloping land can increase the contents of soil pH, total nitrogen (TN), organic carbon (TOC), total sulfur (TS), total phosphorus (TP), and other indicators [12,13]. Cultivating GE also affects the composition of soil microorganisms. The changes in soil microbial communities after GE cultivation using different fungal substrates were analyzed [14], and the results indicated that the abundances of soil bacteria and actinomycetes increased, which improved soil fertility to a certain extent. Another study reported that the abundances of pathogenic fungi such as Fusarium and Ilyonectria increased after GE cultivation [15]. It has also been found that continuous GE cultivation caused significant changes in soil bacterial diversity, with Proteobacteria and Acidobacteria being the dominant phyla [16].

The mechanisms by which GE and *A. mellea* influence soil nutrient levels may include the following aspects. First, the hyphae of *A. mellea* can penetrate and decompose soil organic matter, releasing nutrients such as nitrogen and phosphorus into the surrounding environment, thereby increasing soil nutrient availability [12,13]. Second, the symbiotic interaction between GE and *A. mellea* can alter root exudation patterns, which in turn affects the activity and community structure of rhizosphere microorganisms that play key roles in nutrient cycling [14]. Third, the colonization and metabolic activities of *A. mellea* may modify soil physicochemical properties, such as pH and enzyme activity, indirectly influencing nutrient transformation and mobilization [15,16]. These mechanisms collectively contribute to the observed changes in soil nutrient indicators following GE–*A. mellea* co-cultivation.

Based on the symbiotic characteristics of GE and *A. mellea*, we propose the following hypothesis: *A. mellea* from different sources will significantly affect the yield and accumulation of active components of GE by altering the symbiotic adaptability between the two organisms, thereby exerting an impact on the soil microecology. Specifically, we utilized GE and five distinct *A. mellea* strains from different sources for experimentation. Cultivation trials resembling natural conditions were carried out beneath GE forests in Xiaocaoba, Yiliang, and Wanchang, Zhenxiong, which are key production areas. The aim was to investigate yield and quality discrepancies in Zhaotong GE and their influence on soil environmental factors after co-cultivated with *A. mellea* from different sources. The findings of this research can guide the selection and cultivation of optimal symbiotic *A. mellea* for local GE production in Zhaotong. Additionally, they can facilitate the identification of suitable GE and *A. mellea* symbiotic pairings and support the advancement of the GE industry.

## 2. Results

### 2.1. Comparison of Yield Among GE Co-Cultured with A. mellea of Different Sources

**Figure 1:**
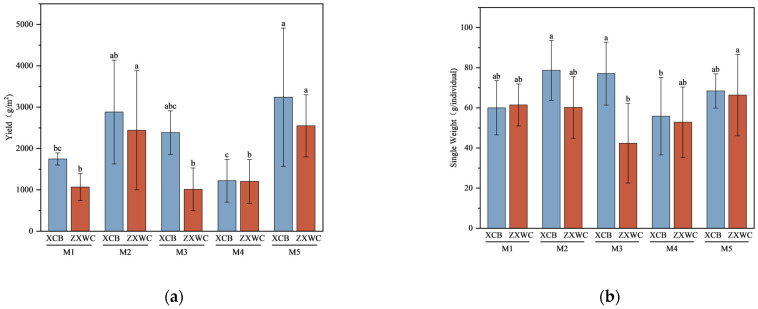
The yield (**a**) and individual weight (**b**) of GE co-cultivated with *A. mellea* of different sources (*n* = 5). a–c: denote groups that differ significantly at the 0.05 level according to Duncan’s test.

The yields and individual weight of GE co-cultivated with five *A. mellea* were analyzed. Results revealed variations in the yield of GE among all groups (Figure 1). In XCB and ZXWC, the yields of GE co-cultivated with various sources of *A. mellea* followed the order M5 > M2 > M3 > M1 > M4. Specifically, at XCB, the yield of GE co-cultivated with M5 was 3239 g/m^2^, markedly surpassing that of those co-planted with M1 and M4. Likewise, at ZXWC, the yield of GE co-cultivated with M5 was 2550 g/m^2^, significantly higher than that of those co-planted with M4, M1, and M3. Notably, the yield of GE at XCB with each *A. mellea* source exceeded the yield observed at ZXWC.

Results revealed variations in the individual weight of GE among all groups (Figure 1). The individual weight of GE co-cultured with different sources of *A. mellea* shows M2 > M3 > M5 > M1 > M4 at XCB, and M5 > M1 > M2 > M4 > M3 at ZXWC. Among them, the individual weight of GE that was co-planted with M2 at XCB was 79 g/individual, which was significantly higher than that of GE co-planted with M4. The individual weight of GE that was co-planted with M5 at ZXWC was 66 g/individual, significantly higher than that of GE co-planted with M3. The individual weight of GE that was co-planted with M2, M3, M4 and M5 at XCB was all higher than that observed at ZXWC.

In conclusion, the yield of GE was the highest when co-cultivated with M5 at both locations.

### 2.2. Analysis of Main Active Components in GE Co-Cultivated with A. mellea of Different Sources

The co-cultivation of *A. mellea* from different sources affected the primary active components of GE, as shown in Table 1. At XCB, the contents of GAS (0.943 mg/g), HBA (1.241 mg/g), PE (1.398 mg/g), PB (5.068 mg/g), PC (1.369 mg/g), and PA (12.174 mg/g) in GE co-cultivated with M1 were significantly higher than those in other treatments. The concentration of PHBA (0.060 mg/g) in GE co-cultivated with M4 was notably higher than that in other treatments, while the content of HBD (0.225 mg/g) was significantly elevated compared with GE treated with M5, M1, and M2.

The content of GAS in GE co-cultivated with M1 at ZXWC (0.925 mg/g) was significantly higher than that in other treatments. The contents of PHBA (0.117 mg/g), PB (5.279 mg/g), PC (1.619 mg/g), and PA (14.002 mg/g) in GE treated with M3 were significantly higher than those in other treatments. The contents of HBA (1.350 mg/g) and PE (1.566 mg/g) in GE co-cultivated with M4 were significantly higher than those in other treatments. The content of HBD in GE treated with M5 (0.236 mg/g) was significantly higher than that in other treatments.

**Figure 2:**
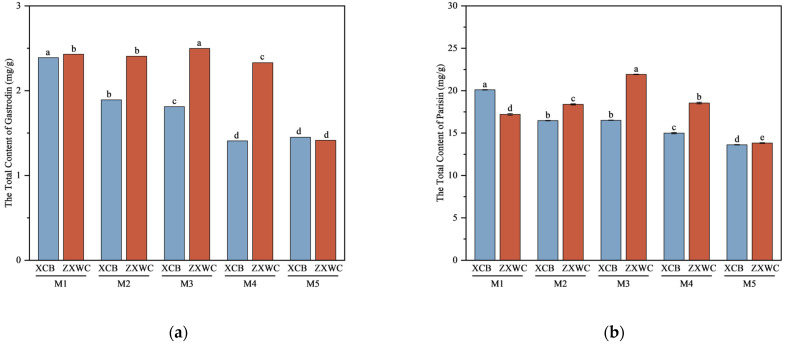
The total content of gastrodin (**a**) and parishin (**b**) of GE co-cultivated with *A. mellea* of different sources (*n* = 3). a–d: denote groups that differ significantly at the 0.05 level according to Duncan’s test.

The total contents of gastrodin and parishin in GE cultivated with *A. mellea* of different sources differed significantly, as shown in Figure 2. The total content of gastrodin was calculated as the sum of four specific phenolic derivatives in GE, namely GAS, HA, PHBA, and HBD. With the exception of GE cultivated with M5, the total gastrodin content at XCB was lower than that at ZXWC. The total content of parishins was determined as the sum of PE, PB, PC, and PA. Similarly, with the exception of GE cultivated with M1, the total content of parishin at XCB was lower than that at ZXWC. Using the total content of gastrodins as the quality evaluation index, the optimal quality of GE was observed in samples from XCB when co-cultivated with M1, and from ZXWC when co-cultivated with M3.

### 2.3. Analysis of Soil Chemical Properties of GE Co-Planted with A. mellea of Different Sources

The chemical properties of GE rhizosphere soil varied significantly with the co-cultivation of *A. mellea* from different sources, as detailed in Table 2. At XCB, soil pH increased significantly after co-cultivation with *A. mellea* from different sources, with the highest value (4.87) recorded in the M4 treatment. Co-cultivation with GE significantly increased soil TOC in M1 (92.31 g/kg) and M2 (83.87 g/kg), but significantly decreased it in M3 (72.26 g/kg) and M4 (64.65 g/kg). For soil TN, a significant increase was observed in M1, M2, M3, and M5, while M4 showed a significant decrease (1.72 g/kg). The soil TP increased significantly in M1 and M5, whereas the soil TK decreased significantly in M4 (9.72 g/kg). The soil AN decreased significantly in M4 (0.22 g/kg) but increased in all other treatments. The soil AP rose significantly in M1 (0.66 mg/kg) and M2 (0.51 mg/kg). Finally, the soil AK increased significantly across all treatments, with the highest value (0.2 g/kg) found in M4.

At ZXWC, following the co-cultivation of GE with *A. mellea* from different sources, soil pH increased significantly across all treatments, with the highest value (5.09) recorded in M4. Total organic carbon (TOC) content in the soil also showed a significant increase in all treatments, peaking in M2 (199.04 g/kg). Total nitrogen (TN) content rose markedly in treatments M1 (2.72 g/kg), M2 (4.81 g/kg), M3 (3.68 g/kg), and M4 (2.87 g/kg), but decreased significantly in M5 (2.52 g/kg). For total phosphorus (TP), a significant increase was observed only in M4 (1.03 g/kg), whereas significant decreases were found in M1 (0.85 g/kg), M3 (0.86 g/kg), and M5 (0.85 g/kg). After co-cultivation with GE, the soil TK content increased significantly in M1 (29.14 g/kg), M3 (29.17 g/kg), M4 (29.92 g/kg), and M5 (30.48 g/kg), but showed a significant decrease in M2 (22.52 g/kg). For soil AN, co-cultivation with GE saw a significant reduction in M3 (0.15 g/kg), whereas all other treatments displayed a significant increase. As for soil AP, a significant decrease was found in M1 (0.69 mg/kg) after co-cultivation with GE, while all other treatments exhibited a significant increase. Finally, the soil AK content was significantly enhanced in all treatments after the co-cultivation of GE with *A. mellea* isolates of different sources, with the highest value (0.32 g/kg) detected in M1.

In summary, the soil pH ranged from 4.67 to 5.09, consistent with the acidic soil criteria outlined in Table S1. This indicates that the soils at both experimental sites are conducive to the growth of GE, which requires a slightly acidic environment (pH 5.0–6.0). Following GE cultivation, the soil pH increased significantly across all treatments. In both experimental sites, co-cultivation with the M2 led to elevated levels of soil TOC, TN, TP, AN, AP, and AK, suggesting that this combination can effectively replenish soil nutrients and enhance soil fertility.

### 2.4. Analysis of Rhizosphere Soil Microbial Community Structure of GE Co-Cultivated with A. mellea from Different Sources

As presented in Table S2, amplon sequencing of the 12 groups of soil samples, followed by filtration, quality control, and splicing, yielded a range of 16S sequences per individual soil sample from 84,370 to 128,637, with a mean of 109,445. A total of 94,271 valid data points were obtained for the bacterial community, resulting in a quality control effectiveness rate of 86.19%. The number of ITS sequences varied from 79,587 to 121,993, with an average of 96,890. For the fungal community, 92,380 valid data points were acquired, achieving a quality control effectiveness rate of 95.36%. The proportion of effective sequences for both bacteria and fungi was relatively high, while chimeric sequences constituted less than 15%. This substantial proportion of valid sequences indicates that the data obtained from this sequencing are appropriate for subsequent investigations into the differences in microbial community structures in the peri-soil of GE co-planted with different *A. mellea*.

#### 2.4.1. OTU Clustering Analysis of Bacterial Communities in the Rhizosphere Soil of GE

**Figure 3:**
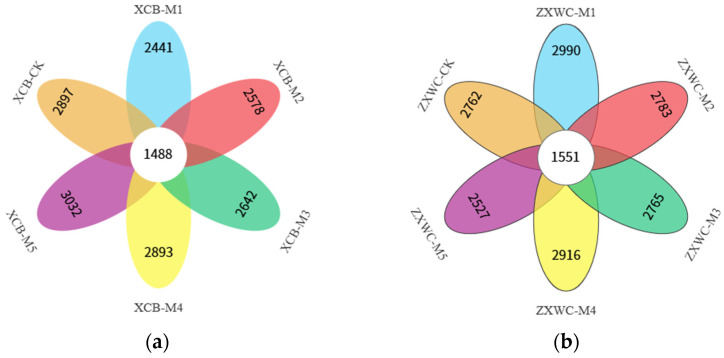
Venn diagrams of soil bacterial OTUs in GE co-planted with *A. mellea* from different sources at XCB (**a**) and ZXWC (**b**) (*n* = 3). XCB-M1~M5: Soil samples from co-cultivation with five *A. mellea* of different sources in XCB; XCB-CK: Soil samples in XCB with no GE–*A. mellea* co-cultivation; ZXWC-M1~M5: Soil samples from co-cultivation with five *A. mellea* of different sources in ZXWC; ZXWC-CK: Soil samples in ZXWC with no GE–*A. mellea* co-cultivation.

The Venn diagrams in Figure 3 illustrate the analysis of soil bacterial species composition similarity and overlap across different treatments. The co-cultivation of *A. mellea* from various sources impacts the soil bacterial community composition in the rhizosphere of GE. Each treatment exhibited a distinct number of specific bacterial OTUs. In the XCB treatments, unique OTUs numbered 2897, 2441, 2578, 2642, 2893, and 3032, respectively. A total of 1448 core otus were identified across all treatments. *A. mellea* of different sources exerted site-specific impacts on soil fungal communities during co-cultivation with GE. At XCB, soil-specific fungal OTUs decreased with M1, M2, M3, and M4 but increased with M5; this trend was reversed at ZXWC.

#### 2.4.2. OTU Clustering Analysis of Fungal Communities in the Rhizosphere Soil of GE

**Figure 4:**
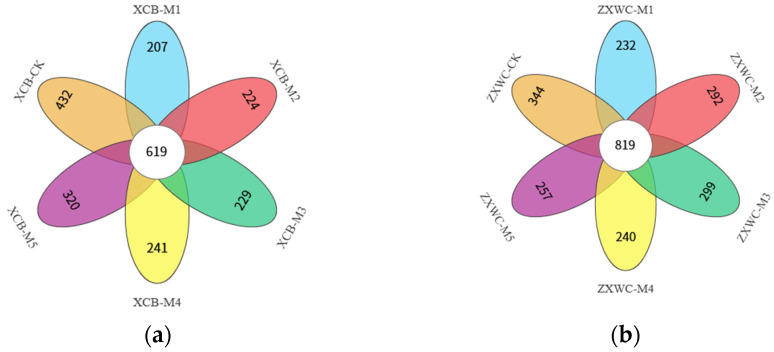
Venn diagrams of soil fungi OTUs in GE co-planted with *A. mellea* of different sources at XCB (**a**) and ZXWC (**b**) (*n* = 3). XCB-M1~M5: Soil samples from co-cultivation with five *A. mellea* of different sources in XCB; XCB-CK: Soil samples in XCB with no GE–*A. mellea* co-cultivation; ZXWC-M1~M5: Soil samples from co-cultivation with five *A. mellea* of different sources in ZXWC; ZXWC-CK: Soil samples in ZXWC with no GE–*A. mellea* co-cultivation.

The Venn diagrams in Figure 4 illustrate the comparison and overlap of soil fungal species composition across various treatments. Co-cultivating *A. mellea* from diverse sources impacts the fungal community composition in the rhizosphere soil of GE. Each treatment exhibited a specific number of unique fungal OTUs. At XCB, there were 432, 207, 224, 229, 241, and 320 unique OTUs, totaling 619 core OTUs across all processes. Similarly, ZXWC displayed 344, 232, 292, 299, 240, and 257 unique OTUs, with a combined total of 819 core OTUs. Soil fungal OTUs were reduced upon co-cultivation with *A. mellea* of different sources.

#### 2.4.3. Analysis of Alpha Diversity of Microbial Communities in the Rhizosphere Soil of GE

The Alpha diversity index of bacteria and fungi in the peri-soil of various GE co-planted strains was assessed through statistical single-factor analysis, presented in Table 3 and Table 4. Higher Shannon and Simpson indices indicate increased species diversity within the microbial community. The Chao1 and ACE indices were utilized to evaluate species richness among soil microbial communities subjected to different strain treatments. Examination of the Goods_coverage index for the soil microbial community surrounding GE revealed coverage ranging from 95.39% to 99.56% across all samples, suggesting a low probability of undetected sequences in the samples. The sequencing outcomes effectively depicted the diversity of the soil microbial community surrounding GE co-cultivated with *A. mellea* from different sources (*p* < 0.05).

In terms of bacterial diversity, at XCB, the soil bacterial Shannon index increased after co-cultivation of GE with *A. mellea* of different sources, being significantly elevated with M2 (9.76), M3 (9.82), M4 (9.68), and M5 (9.84). The soil bacterial Simpson index remained unchanged at 0.99 across all treatments. At ZXWC, the soil bacterial Shannon index rose after co-cultivation with GE and M2 (10.20), M3 (10.17), M4 (10.06), and M5 (10.15), though not significantly; the soil bacterial Simpson index was stable at 0.99 for all treatments. For bacterial richness, the soil bacterial Chao1 and Ace indices increased at XCB but decreased at ZXWC after co-cultivation with *A. mellea* of different sources, with no significant differences observed in either case.

In terms of fungal diversity, at XCB, the soil fungal Shannon index decreased significantly after co-cultivation of GE with *A. mellea* of different sources. The soil fungal Simpson index decreased with M1 (0.96) and M5 (0.96), but not significantly. At ZXWC, the soil fungal Shannon index decreased significantly after co-cultivation of GE with M1 (6.49), M2 (6.95), M3 (6.34), and M4 (6.34), and the soil fungal Simpson index decreased across all treatments with no significant differences. In terms of fungal richness, at XCB, the soil fungal Chao1 index increased with M2 (2349.63), M3 (2292.19), M4 (2279.25), and M5 (2184.60), with the Chao1 and Ace indices significantly elevated with M2. At ZXWC, the soil fungal Chao1 and Ace indices decreased after co-cultivation with *A. mellea* of different sources, with no significant differences.

#### 2.4.4. Analysis of Bacterial and Fungal Community Structure in the Rhizosphere Soil of GE

**Figure 5:**
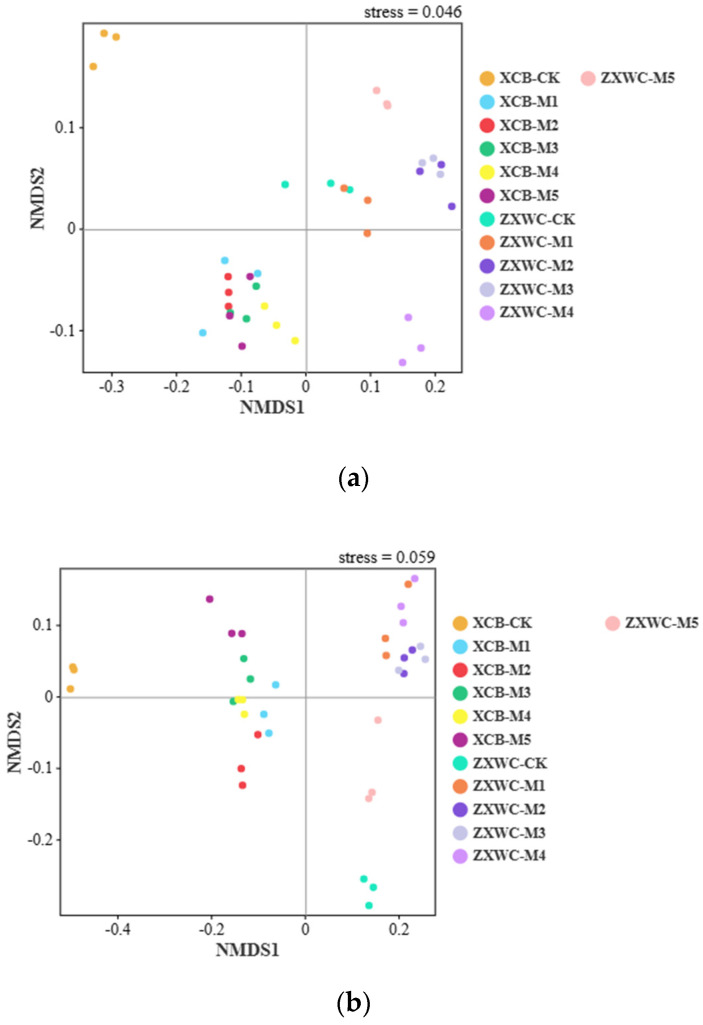
NMDS analysis of soil bacterial (**a**) and fungal (**b**) communities under co-cultivation of GE with *A. mellea* from different sources (n = 3). XCB-M1~M5: Soil samples from co-cultivation with five *A. mellea* of different sources in XCB; XCB-CK: Soil samples in XCB with no GE–*A. mellea* co-cultivation; ZXWC-M1~M5: Soil samples from co-cultivation with five *A. mellea* of different sources in ZXWC; ZXWC-CK: Soil samples in ZXWC with no GE–*A. mellea* co-cultivation.

Non-metric multidimensional scaling (NMDS) was employed to analyze the effects of co-cultivating GE with *A. mellea* from different sources on the structure of soil bacterial and fungal communities, with results presented in Figure 5. The stress values of the bacterial and fungal ordination models were 0.046 and 0.059, respectively, both of which were well below the threshold of 0.2, indicating that the ordination results were reliable and could accurately reflect differences in community composition.

Moreover, significant microbial segregation was observed among GE co-planted with *A. mellea* of different sources at both XCB and ZXWC. At XCB, the control (XCB-CK) formed an independent cluster clearly distinct from all inoculated treatments (XCB-M1, M2, M3, M4, and M5) for both bacteria and fungi, demonstrating that inoculation with *A. mellea* significantly modified the soil microbial communities. At ZXWC, ZXWC-M5 formed a unique fungal cluster separated from the other treatments, and a similar differentiation pattern was also detected in the bacterial community. Collectively, both the cultivation site and *A. mellea* were key factors affecting soil bacterial and fungal communities during GE cultivation, and the effect of the site was stronger than that of the inoculated *A. mellea*.

#### 2.4.5. Analysis of Microbial Species and Their Relative Abundances in the Rhizosphere Soil of GE

**Figure 6:**
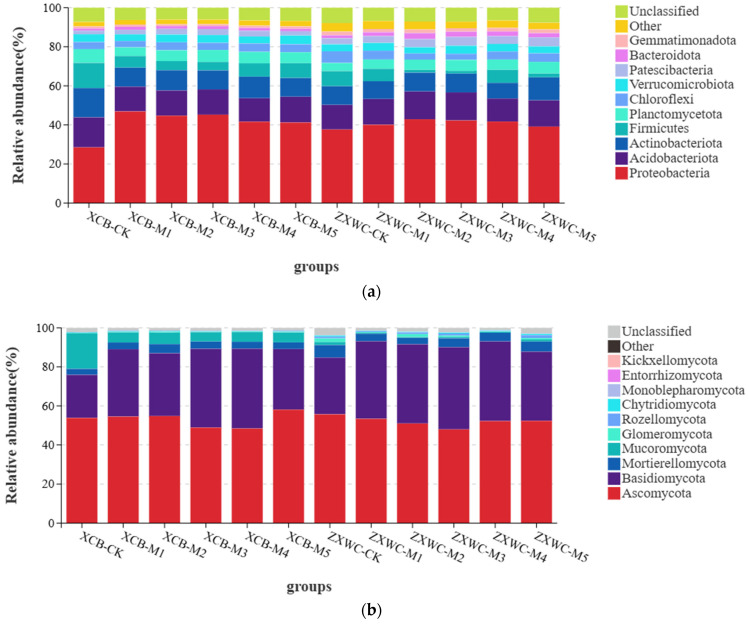
Relative abundances of rhizosphere bacteria (**a**) and fungi (**b**) of GE (n = 3). XCB-M1~M5: Soil samples from co-cultivation with five *A. mellea* of different sources in XCB; XCB-CK: Soil samples in XCB with no GE–*A. mellea* co-cultivation; ZXWC-M1~M5: Soil samples from co-cultivation with five *A. mellea* of different sources in ZXWC; ZXWC-CK: Soil samples in ZXWC with no GE–*A. mellea* co-cultivation.

No significant differences were observed in the soil bacterial community at the phylum level following the co-cultivation of GE with *A. mellea* from various sources; however, notable differences in relative abundance were detected in Figure 6a. The ten dominant bacterial phyla in the rhizosphere soil of GE co-planted with different *A. mellea*, ranked by relative abundance, was consistent across samples. These phyla, listed from highest to lowest abundance, include *Proteobacteria* (28.50–46.88%), *Acidobacteriota* (11.78–15.36%), *Actinobacteriota* (8.02–15.00%), Firmicutes (1.37–12.85%), *Planctomycetota* (4.15–7.09%), *Chloroflexi* (3.11–5.97%), *Verrucomicrobiota* (3.17–4.48%), *Patescibacteria* (1.85–4.63%), *Bacteroidota* (1.09–3.09%), and *Gemmatimonadota* (0.95–1.91%). Collectively, these phyla account for 89.96% of the total bacterial sequences, as illustrated in Figure 5. *Proteobacteria* dominated each sample, representing the largest proportion of bacteria. *Acidobacteriota* and *Actinobacteriota* followed as the second and third most prevalent bacterial groups, respectively. At both XCB and ZXWC, soil *Proteobacteria* abundance increased after co-cultivation of GE with *A. mellea* of different sources. At XCB, the highest *Proteobacteria* abundance was seen with M1 (46.88%), and at ZXWC, with M2 (42.88%). At XCB, soil *Acidobacteria* abundance decreased across all treatments, and *Proteobacteria* abundance was lowest with M4 (12.01%). At ZXWC, *Proteobacteria* abundance increased with M1, M2, M3 and M5, but decreased with M4. At XCB, soil *Actinobacteria* abundance decreased across all treatments, with the lowest value (9.59%) for M5. At ZXWC, *Actinobacteria* abundance decreased with M1 and M4, increased with M2, M3, and M5, and peaked with M5 (11.78%).

There was no significant difference in the soil bacterial community at the genus level following the co-cultivation of GE with *A. mellea* from various sources; however, a notable variance existed in relative abundance in Figure 6b. The prevalent fungal genera in the soil of each treatment were *Massilia* (2.58–15.98%), *Burkholderia-caballerania-paraburkholderia* (5.22–15.18%), *Bacillus* (0.61–5.41%), *Tumebacillus* (0.38–11.74%), *Candidatus_Solibacter* (1.47–2.12%), *Bradyrhizobium* (1.03–1.92%), *Acidothermus* (0.87–1.83%), *Bryobacter* (0.82–1.67%), *Acidibacter* (0.69–1.37%), and *HSB_OF53-F07* (1.61–3.59%).

At XCB, soil *Ascomycota* abundance increased following co-cultivation of GE with M1, M2, and M5, but decreased with M3 and M4. At ZXWC, soil *Ascomycota* abundance declined across all treatments involving co-cultivation of GE with *A. mellea* isolates of different sources, with the lowest value observed for M3 (45.61%). At both XCB and ZXWC, the abundance of soil *Basidiomycota* increased after co-cultivation of GE with *A. mellea* of different sources. Specifically, the highest abundance of soil *Basidiomycota* was recorded with M4 (40.28%) at XCB and with M3 (40.10%) at ZXWC. At XCB, the abundance of soil *Mortierellomycota* increased across all treatments, reaching the highest value with M2 (4.35%); at ZXWC, it decreased across all treatments, with the lowest value found for M2 (3.15%).

#### 2.4.6. Functional Prediction of the Rhizosphere Soil Microbial Community of GE

**Figure 7:**
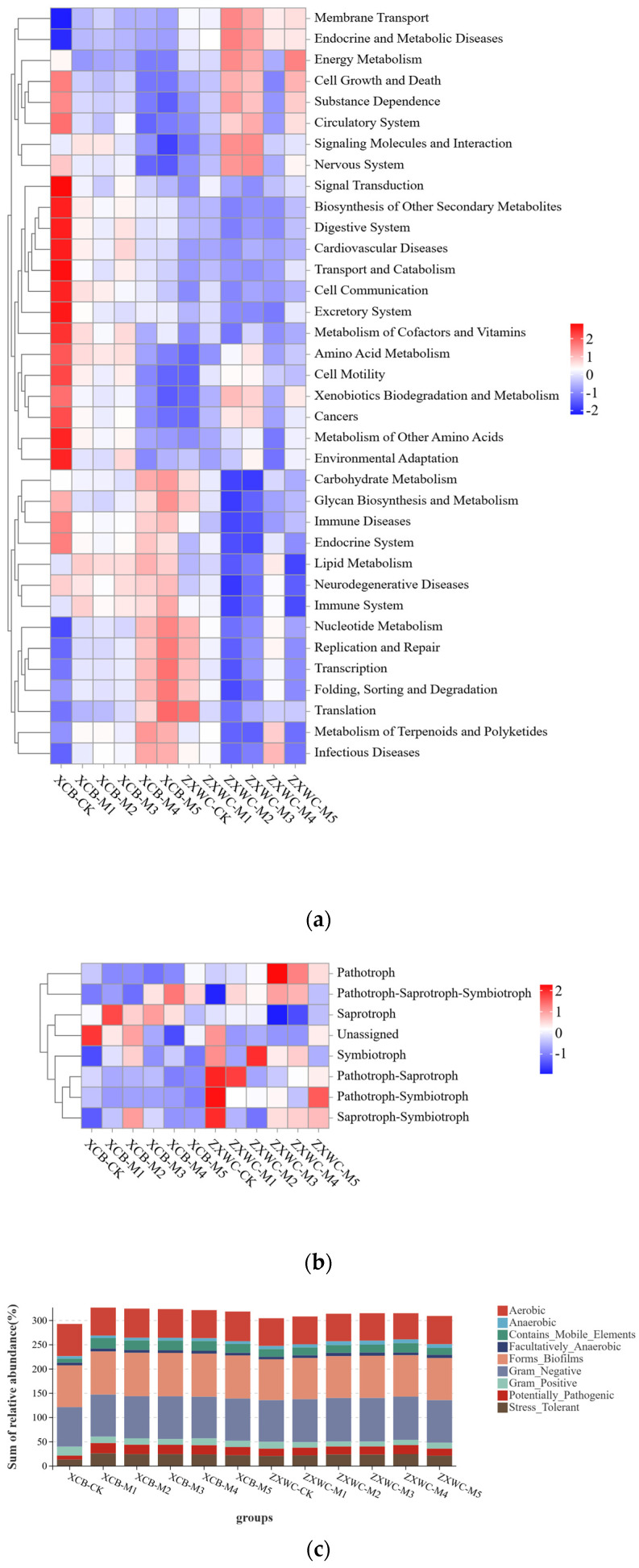
Functional analysis of the rhizosphere soil microbial community of GE (n = 3). (**a**) Clustering of relative abundances of functional annotations for bacteria in the rhizosphere soil of GE. (**b**) Clustering of relative abundances of functional annotations for fungi in the rhizosphere soil of GE. (**c**) Phenotypic abundance of the bacterial community in the rhizosphere soil of GE. XCB-M1~M5: Soil samples from co-cultivation with five *A. mellea* of different sources in XCB; XCB-CK: Soil samples in XCB with no GE–*A. mellea* co-cultivation; ZXWC-M1~M5: Soil samples from co-cultivation with five *A. mellea* of different sources in ZXWC; ZXWC-CK: Soil samples in ZXWC with no GE–*A. mellea* co-cultivation.

Phenotypic prediction of bacterial communities in the rhizosphere soil of GE was conducted using Bug Base, and the phenotypic abundances following co-cultivation with *A. mellea* isolates of different origins are presented in Figure 7a. At both XCB and ZXWC, among the three phenotypes (aerobic, anaerobic, and facultatively anaerobic), the relative abundance of aerobic bacteria was higher than that of anaerobic and facultatively anaerobic bacteria in all soil samples, including the initial soil before GE planting and soils after co-cultivation with *A. mellea* of different sources. At XCB, the abundance of aerobic bacteria decreased following co-cultivation of GE with *A. mellea* of different sources. At both sites, the abundance of bacteria associated with biofilm formation increased after co-cultivation with *A. mellea* of different sources. Similarly, the abundance of bacteria harboring mobile elements increased in soils co-cultivated with M1, M2, M3, and M4 at both XCB and ZXWC. Furthermore, at both sites, the abundance of Gram-negative bacteria increased and reached the maximum with M3, whereas the abundance of Gram-positive bacteria decreased and reached the minimum with M3.

Fungal communities in the rhizosphere soil of GE were analyzed using FUN Guild, through which the taxonomic composition, abundance, and ecological functions were determined in Figure 7b. A total of seven fungal trophic modes were identified in soils after co-cultivation of GE with *A. mellea* of different sources, among which the *Pathotroph–Saprotroph–Symbiotroph* style was ecologically dominant. At XCB, the dominant fungi in the control soil (CK, without GE planting) were Unassigned. The dominant fungi in soil co-cultivated with M1 were Saprotrophs. Soils co-cultivated with M2 were dominated by Saprotroph–Symbiotroph and Unassigned fungi. Soils co-cultivated with M3 were dominated by Saprotrophs, while those co-cultivated with M4 were dominated by the *Pathotroph–Saprotroph–Symbiotroph* trophic mode. At ZXWC, the dominant fungi in the CK were Unassigned, *Pathotroph–Saprotroph*, *Pathotroph–Symbiotroph*, and *Saprotroph–Symbiotroph*. Soil co-cultivated with M1 was dominated by *Saprotrophs*. Soil co-cultivated with M2 was dominated by *Pathotroph–Saprotroph*. Soil co-cultivated with M3 was dominated by *Pathotroph* and *Pathotroph–Saprotroph–Symbiotroph*. Soil co-cultivated with M4 was dominated by *Pathotroph*. Soil co-cultivated with M5 was dominated by *Pathotroph–Symbiotroph*.

Bacterial communities in the rhizosphere soil of GE were analyzed using Tax4Fun, and a clustering heatmap showing the top 35 KEGG metabolic pathways at Level 2 was constructed in Figure 7c. The dominant metabolic pathways of rhizosphere bacteria after co-cultivation of GE with *A. mellea* of different sources included carbohydrate metabolism and amino acid metabolism in Table S3. As shown in Figure 7c, at XCB, bacteria in the CK were enriched in pathways including signal transduction, digestive system, cardiovascular diseases, transport and catabolism, and cell communication.

## 3. Discussion

The results of this study indicate that the effects of *A. mellea* from different sources on the yield and quality of GE vary. GE achieved the highest yield when co-cultivated with M5. This result, in which GE attained the maximum yield, is in agreement with earlier observations [17], confirming the critical role of strain-specificity of *A. mellea* on GE productivity. From the perspective of CSR theory (Competitor–Stress Tolerator–Ruderal theory), GE co-cultivated with M5 may exhibit a more R-selected (ruderal) growth pattern, allocating more of the available resources to tuber expansion at the expense of secondary metabolite accumulation under resource-sufficient conditions. In contrast, the highest total contents of parishin and gastrodin were observed with M1 at XCB and M3 at ZXWC, suggesting that these combinations induce a more S-selected (stress-tolerance) metabolic response, diverting resources toward the synthesis of defensive compounds. Previous studies [18,19,20] have also shown that the growth characteristics of different *A. mellea* strains affect GE quality, which is consistent with the findings of this study.

Since GE remains subterranean for most of its life cycle [21], its growth inevitably influences soil nutrient composition. In this study, soil pH values increased significantly following cultivation with *A. mellea* from different sources. Soil nutrient indices were elevated after cultivation with M1 and M2 at XCB and with M4 at ZXWC, which aligns with findings reported in previous studies [13,21,22]. One possible explanation is that *A. mellea*, through the secretion of organic acids and extracellular enzymes, both decomposes soil organic matter to release nutrients and alters the acid–base balance of the rhizosphere microenvironment. Notably, M4 led to a reduction in soil nutrients at both XCB and ZXWC, suggesting that not all *A. mellea* strains possess the ability to mobilize nutrients; conversely, some strains may negatively impact GE by competing for limiting soil nutrients. This finding underscores the importance of strain selection and suggests that the effect of *A. mellea* on soil nutrients is strain-dependent rather than a general characteristic of the genus. Hyphal colonization can promote the formation of organic carbon [23]. In this study, soil TOC content increased after cultivation with M1 and M2 at both the ZXWC and XCB, indicating that both M1 and M2 exhibited a capacity to regulate soil acidity and enhance carbon sequestration. However, further research is needed to determine whether this effect is driven by direct contributions of fungal biomass to the organic carbon pool or by indirect stimulation of root exudates.

The impact of different *A. mellea* sources on the soil microbial community during GE cultivation showed strain-specific and site-dependent characteristics. In this study, bacterial diversity increased while fungal diversity decreased in GE soils from both sites after cultivation with *A. mellea* from different sources, consistent with the results of earlier observations [15]. This pattern can be explained by niche theory: after colonization, *A. mellea* suppresses certain fungal groups through resource competition and niche occupation, while simultaneously creating a more favorable microenvironment for bacteria (e.g., altered pH, nutrient availability), thereby promoting bacterial proliferation and differentiation [24]. The general reduction in the number of unique fungal OTUs further supports the consistent inhibitory effect of *A. mellea* on fungal communities. However, an important alternative explanation is that GE itself may directly shape the rhizosphere microbial community through root exudates, rather than *A. mellea* acting alone. Previous studies have demonstrated that plants can significantly influence soil microbial community composition by secreting root exudates, which either attract or deter specific microbial taxa [25]. Therefore, it is difficult to attribute the observed microbial changes exclusively to either GE or *A. mellea*. Instead, these changes may represent an emergent property of the GE and *A. mellea* holobiont, potentially shaped by GE root exudates and *A. mellea*-mediated modifications to the rhizosphere microenvironment. The changes in unique bacterial OTUs varied by site: at the Xiaocaoba site, unique bacterial OTUs decreased after cultivation with M1, M2, M3, and M4 but increased with M5, while the opposite trend was observed at ZXWC. This site-specificity likely reflects differences in baseline soil conditions and native microbial communities between the two locations, which may modulate host–symbiont–microbiome interactions at the local scale. These findings are similar to those reported in related studies [23,26,27]. Compared to soils without GE cultivation, both bacterial and fungal OTUs decreased in the rhizosphere soils of GE, indicating that GE cultivation disrupts the balance of soil microorganisms, consistent with findings from previous reports [28].

The colonization of different *A. mellea* sources promoted beneficial soil microorganisms to varying extents. Although no significant differences were observed at the phylum level, relative abundances varied notably among treatments. This suggests that *A. mellea* primarily modulates the population sizes of existing microbial groups rather than introducing new taxa—a pattern consistent with the ecological concept of the “priority effect,” whereby early colonizers shape community assembly by modifying environmental conditions [29]. Across both sites, *Proteobacteria*, *Acidobacteria*, and *Actinobacteria* were the dominant bacterial phyla. *Proteobacteria* abundance increased after *A. mellea* cultivation, consistent with previous reports [23]. Given that *Proteobacteria* are copiotrophic and thrive under nutrient-rich conditions, this increase may indirectly reflect *A. mellea*-mediated enhancement of soil nutrient availability. This interpretation is supported by the well-established roles of Proteobacteria in nitrogen cycling and organic matter degradation [30,31], aligning with the elevated nitrogen and organic matter content observed in this study. Notably, only M4 reduced the abundances of *Acidobacteria* and *Actinobacteria* at both sites, suggesting that this strain may negatively affect these ecologically important groups—potentially explaining the lower nitrogen and organic matter content in the M4 treatment. However, whether the decline of these phyla directly causes nutrient reduction or is a consequence of lower nutrient availability remains correlational rather than causal.

Functional prediction analysis of soil microorganisms revealed that cultivation with *A. mellea* from different sources resulted in an increase in predicted Gram-negative bacteria and a decrease in predicted Gram-positive bacteria. It must be emphasized that the Gram typing provided by BugBase is a prediction based on 16S rRNA gene sequences, not direct Gram staining results. Therefore, these findings should be interpreted as inferred functional potential rather than confirmed phenotypic traits. Previous studies have shown that Gram-negative bacterial abundance is positively correlated with soil organic matter content [32]. If this association were causal, it could explain the highest organic matter content in the M3 treatment, which also exhibited the highest Gram-negative and lowest Gram-positive abundance. However, due to the correlational design of this study, we cannot determine whether increased organic matter is a cause or a consequence of Gram-negative bacterial proliferation. Further validation is therefore needed in future studies.

Although this study revealed differential effects of *A. mellea* from different sources on the yield, quality, and rhizosphere soil microecology of GE, several limitations should be acknowledged. First, this study lacked direct analyses of the genomic characteristics and secreted metabolites of *A. mellea* itself; consequently, whether genetic differences or geographic origin-related divergence exist among the tested strains remains unclear. Second, our conclusions are primarily host-centered, as the mechanisms of *A. mellea* action were inferred indirectly through host growth responses, active compound accumulation, and changes in rhizosphere microbial communities, without direct characterization of the biological properties of *A. mellea*. Therefore, the mechanisms underlying strain-specific differences and their ecological effects are only indirectly supported. Future studies should integrate fungal omics approaches to directly analyze the genetic basis and functional differences in *A. mellea* from different sources from the perspective of the fungus itself, thereby providing a more comprehensive understanding of the interaction mechanisms within the GE–*A. mellea* symbiotic system.

## 4. Materials and Methods

### 4.1. Overview of the Test Site

As presented in Table 5, amplicon sequencing of 12 groups of soil samples, followed by filtration, quality control, and splicing, yielded a range of 16S sequences per individual soil sample from 84,370 to 128.

### 4.2. Test Materials

The tested seeds were Gastrodia elata f. *glauca* cultivated and produced by Yunnan Senhao Fungus Industry Co., Ltd., Zhaotong. Five distinct sources of *A. mellea*, typically employed in the cultivation and production of GE in Zhaotong, were chosen to be co-cultivated with GE in Table 6.

### 4.3. Experimental Design

The experiment was arranged in a randomized complete block design (RCBD); five distinct strains of *A. mellea* sourced from various origins were utilized to establish fungus beds at the aforementioned pair of experimental locations in September 2022. Each strain of *A. mellea* was allocated five beds at separate experimental sites, while GE was omitted to serve as the control. The pond dimensions were set at 50 cm in width by 100 cm in length, with a depth of 20 cm. The fungal substrates consisted of sections of chestnut trees measuring 5 to 10 cm in diameter and 20 to 30 cm in length. Each pond received 15 kg of fungal substrates, evenly distributed at the pond’s base. A quantity of 1 kg of *A. mellea* was allocated for each pond, broken into approximately 5 cm sized fragments and positioned in close proximity to the fungal substrates. The setup was completed by covering the materials with about 5 to 10 cm of soil, ensuring that the soil cover height slightly exceeded that of the surrounding ground. In March 2023, the topsoil of the pond was excavated and planted with 20 pieces (approximately 200 g) of white-headed hemp per pond, accompanied by 1.5 kg of fungal materials. All other planting practices in each plot were kept consistent, except for the different sources of *A. mellea*.

### 4.4. Sample Collection and Processing

In November 2023, samples of GE and soil were collected and pretreated. The yield of GE was recorded as fresh weight (g/m^2^). After determining the yield, five uniform-sized, healthy, and undamaged GE tubers were randomly selected from each treatment for component analysis. The GE samples were washed clean, steamed until just cooked through, dried at 50 °C to constant weight, ground, and passed through a 60-mesh sieve for the determination of chemical components.

Rhizosphere soil was defined as soil firmly adhering to the root surface. To obtain representative samples, five sampling points were established per treatment as spatial replicates. At each sampling point, the five-point sampling method [33] was used to collect GE rhizosphere soil as follows: five GE plants were selected, the tubers were carefully excavated, and the soil tightly adhering to the root surface was collected and pooled to form a composite sample for that sampling point. The five composite samples from the five sampling points were then mixed in equal amounts to form one representative mixed sample per treatment. Three such independent mixed samples were prepared per treatment as biological replicates (n = 3). Control soil was collected from the same field site but from areas not subjected to the GE and *A. mellea* co-cultivation trial, using the same sampling method as described above.

Fresh soil samples for microbial analysis were immediately frozen at −86 °C until DNA extraction. The remaining soil was air-dried and sieved through a 100-mesh sieve for subsequent chemical analyses. Due to differences in baseline soil properties, GE and soil samples from the two field sites were analyzed separately, and the site was treated as a fixed factor in subsequent combined analyses.

### 4.5. Determination Method

#### 4.5.1. Determination of Active Ingredients in GE

The liquid sample was prepared by weighing 1.0 g of GE powder, adding 20 mL of 75% ethanol, and conducting ultrasonic extraction for 30 min. The solution was filtered through a 0.22 μm filter and stored for future use.

Chromatographic analysis was performed using a Welchrom C18 column (4.6 mm × 250 mm, 5 μm) from Yuexu Technology. The mobile phase consisted of acetonitrile (B) and a 0.05% phosphoric acid solution (A). The flow rate was set at 1 mL∙min^−1^, with detection at a wavelength of 270 nm and a column temperature of 30 °C. The injection volume was 10 μL, and the gradient elution times are detailed in Table 7.

Calibration curves for the eight target compounds were established by plotting peak area against concentration. Linearity was assessed using the correlation coefficient (R^2^). Spiked recovery experiments were conducted in triplicate (n = 3) to evaluate accuracy and precision. The regression equations, linear ranges, R^2^ values, average recoveries, and RSD for all eight compounds are summarized in Table 8.

#### 4.5.2. Determination of Soil Chemical Properties

The pH, total organic carbon (TOC), total nitrogen (TN), total phosphorus (TP), total potassium (TK), alkali-hydrolyzable nitrogen (AN), available phosphorus (AP), and available potassium (AK) of the rhizosphere soil of *Gastrodia elata* (GE) and the control group were analyzed following established methods from the literature [23,34,35]. All soil analyses were conducted by Nanjing Kavins Testing Technology Co., Ltd. (Nanjing, China).

#### 4.5.3. Soil Microbial Sequencing

Soil DNA was extracted using the HiPure Soil DNA Extraction Kit (Magen, Guangzhou, China) following the manufacturer’s instructions. For quality control, three technical replicates were performed per sample, and negative controls (no soil template) were included in each extraction batch to monitor cross-contamination.

The V3–V4 region of the bacterial 16S rRNA gene was amplified using barcoded primers 341F (5′-CCTACGGGNGGCWGCAG-3′) and 806R (5′-GGACTACHVGGGTATCTAAT-3′). The fungal ITS2 region was amplified using barcoded primers ITS3_KYO2 (5′-GATGAAGAACGYAGYRAA-3′) and ITS4 (5′-TCCTCCGCTTATTGATATGC-3′). PCR amplification was performed in a 50 μL reaction system containing: 10 μL of 5× Q5^®^ Reaction Buffer (New England Biolabs, Ipswich, MA, USA), 10 μL of 5× Q5^®^ High GC Enhancer (New England Biolabs, Ipswich, MA, USA), 1.5 μL of 2.5 mM dNTPs, 1.5 μL each of forward and reverse primers (10 μM), 0.2 μL of Q5^®^ High-Fidelity DNA Polymerase (New England Biolabs, Ipswich, MA, USA), 50 ng of template DNA, and ddH_2_O to volume. The PCR conditions were as follows: initial denaturation at 95 °C for 5 min; 30 cycles of denaturation at 95 °C for 1 min, annealing at 60 °C for 1 min, and extension at 72 °C for 1 min; followed by a final extension at 72 °C for 7 min. Three PCR replicates were performed per sample, which were then pooled prior to sequencing. All PCR reagents were sourced from New England Biolabs, Ipswich, MA, USA.

Sequencing libraries were constructed using the Illumina DNA Prep Kit (Illumina, San Diego, California, USA), and library quality was assessed using the ABI StepOnePlus Real-Time PCR System (Thermo Fisher Scientific, Waltham, MA, USA). The amplicons were purified, quantified, and sequenced on the Novaseq 6000 platform (Illumina, San Diego, CA, USA) using the PE250 mode. Sequence data were quality-filtered, and chimeras were removed using standard pipelines in QIIME2 v2022.2. The sequencing work was completed by Guangzhou Genedenovo Biotechnology Co., Ltd., Guangzhou, China.

### 4.6. Data Processing and Analysis

Data statistics and analysis were performed using Excel 2021, DPS 9.01, and SPSS 21.0. Plots were generated with Origin Pro 2025, while the analysis of soil microbial community structure and diversity was conducted on the Genedenovo Biological Cloud platform.

The precision of reported values was determined by the detection limits and measurement capabilities of the respective instruments. Yield data (fresh weight) were recorded to the nearest gram and reported as whole numbers (g/m^2^). Concentrations of active compounds determined by HPLC were reported to three decimal places (mg/g), reflecting the instrument’s standard output. Soil chemical parameters were reported to two decimal places, consistent with the resolution of the analytical methods employed. All rounding followed standard rules.

## 5. Conclusions

This study compared the differences in yield, quality, and soil environmental factors of GE cultivated with *A. mellea* from different sources. Based on our one-year field trial, GE co-cultivated with M5 showed the highest yield. However, given the site-dependent effects observed, this recommendation should be considered preliminary, and long-term, multi-site trials are needed before general recommendations can be made. The results indicate that M1 at XCB and M3 at ZXWC are conducive to producing high-quality GE. Therefore, cultivation strategies can be tailored to specific needs, such as high yield, superior quality, medicinal use, culinary use, or dual-purpose applications. Cultivation with *A. mellea* from different sources enhanced soil nutrients, but the regulatory effects were both site- and strain-specific. Precise strain selection can optimize the soil microenvironment for GE cultivation. Further analysis of the functional mechanisms of key microbial groups will provide valuable insights for high-quality GE cultivation and targeted regulation of microbial communities. This study has several limitations. The conclusions were drawn indirectly with the host (GE) as the focus, and the *A. mellea* were not identified to clarify potential genetic differences. Future research should integrate fungal omics analyses for a more in-depth investigation.

## Figures and Tables

**Table 1:** Content of main active components (mg/g) of GE after co-cultivation with *A. mellea* of different sources ($\bar{x}\;$± s, n = 3).

Experimental Variants	GAS	HBA	PHBA	HBD	PE	PB	PC	PA
XCB-M1	0.943 ± 0.036 a	1.241 ± 0.008 a	0.017 ± 0.001 d	0.188 ± 0.000 b	1.398 ± 0.007 a	5.068 ± 0.009 a	1.469 ± 0.008 a	12.174 ± 0.012 a
XCB-M2	0.738 ± 0.028 b	0.940 ± 0.001 c	0.036 ± 0.002 b	0.178 ± 0.000 c	0.970 ± 0.011 d	4.935 ± 0.009 b	1.252 ± 0.004 c	9.313 ± 0.011 c
XCB-M3	0.568 ± 0.030 c	0.993 ± 0.003 b	0.028 ± 0.008 c	0.223 ± 0.001 a	1.218 ± 0.003 b	4.669 ± 0.009 c	1.259 ± 0.009 c	9.369 ± 0.016 b
XCB-M4	0.449 ± 0.015 d	0.673 ± 0.003 e	0.060 ± 0.000 a	0.225 ± 0.003 a	1.088 ± 0.006 c	4.078 ± 0.027 e	1.292 ± 0.010 b	8.541 ± 0.038 d
XCB-M5	0.444 ± 0.019 d	0.775 ± 0.003 d	0.041 ± 0.001 b	0.190 ± 0.001 b	0.918 ± 0.002 e	4.302 ± 0.017 d	1.100 ± 0.009 d	7.298 ± 0.043 e
ZXWC-M1	0.925 ± 0.015 a	1.298 ± 0.004 c	0.020 ± 0.001 d	0.187 ± 0.001 b	1.361 ± 0.015 b	4.605 ± 0.058 c	1.375 ± 0.006 d	9.865 ± 0.028 d
ZXWC-M2	0.870 ± 0.019 b	1.323 ± 0.002 b	0.022 ± 0.001 d	0.190 ± 0.001 b	1.083 ± 0.003 c	5.179 ± 0.020 b	1.499 ± 0.010 b	10.625 ± 0.044 c
ZXWC-M3	0.872 ± 0.005 b	1.322 ± 0.001 b	0.117 ± 0.000 a	0.186 ± 0.001 b	1.025 ± 0.002 d	5.279 ± 0.016 a	1.619 ± 0.002 a	14.002 ± 0.017 a
ZXWC-M4	0.772 ± 0.020 c	1.350 ± 0.006 a	0.046 ± 0.002 c	0.162 ± 0.002 c	1.566 ± 0.003 a	4.393 ± 0.030 d	1.439 ± 0.009 c	11.143 ± 0.068 b
ZXWC-M5	0.400 ± 0.007 d	0.727 ± 0.003 d	0.052 ± 0.001 b	0.236 ± 0.004 a	0.899 ± 0.003 e	4.447 ± 0.014 d	1.292 ± 0.007 e	7.191 ± 0.037 e

Different lowercase letters within the same column indicate significant differences according to Duncan’s test (*p* < 0.05). XCB-M1~M5: GE samples from co-cultivation with five *A. mellea* of different sources in XCB; ZXWC-M1~M5: GE samples from co-cultivation with five *A. mellea* of different sources in ZXWC. GAS: gastrodin; HBA: *p*-Hydroxybenzyl alcohol; PHBA: *p*-Hydroxybenzoic acid; HBD: *p*-Hydroxybenzaldehyde; PE: Parishin E; PB: Parishin B; PC: Parishin C; PA: Parishin A.

**Table 2:** Changes in soil chemical properties after GE co-cultivation with *A. mellea* of different sources ($\bar{x}$ ± s, n = 3).

Experimental Variants	PH	TOC (g/kg)	TN (g/kg)	TP (g/kg)	TK (g/kg)	AN (g/kg)	AP (mg/kg)	AK (g/kg)
XCB-CK	4.28 ± 0.01 d	78.10 ± 0.63 c	1.88 ± 0.00 e	0.51 ± 0.01 b	10.22 ± 0.09 ab	0.24 ± 0.00 d	0.29 ± 0.03 cd	0.08 ± 0.00 e
XCB-M1	4.71 ± 0.01 b	92.31 ± 1.36 a	2.60 ± 0.00 b	0.64 ± 0.01 a	10.54 ± 0.09 a	0.31 ± 0.00 a	0.66 ± 0.07 a	0.18 ± 0.00 c
XCB-M2	4.67 ± 0.02 c	83.87 ± 1.16 b	3.19 ± 0.00 a	0.53 ± 0.03 b	10.55 ± 0.33 a	0.28 ± 0.00 b	0.51 ± 0.06 b	0.19 ± 0.00 b
XCB-M3	4.73 ± 0.01 b	72.26 ± 1.45 d	1.94 ± 0.01 d	0.52 ± 0.02 b	9.94 ± 0.15 bc	0.25 ± 0.00 c	0.32 ± 0.03 cd	0.18 ± 0.00 c
XCB-M4	4.87 ± 0.01 a	64.65 ± 1.55 e	1.72 ± 0.01 f	0.51 ± 0.01 b	9.72 ± 0.32 c	0.22 ± 0.01 e	0.36 ± 0.02 c	0.20 ± 0.00 a
XCB-M5	4.86 ± 0.05 a	77.63 ± 0.86 c	2.28 ± 0.01 c	0.63 ± 0.02 a	10.30 ± 0.15 ab	0.28 ± 0.00 b	0.25 ± 0.03 d	0.16 ± 0.00 d
ZXWC-CK	4.75 ± 0.02 c	84.15 ± 0.84 f	2.68 ± 0.01 e	0.91 ± 0.02 b	28.50 ± 0.43 c	0.29 ± 0.00 e	1.09 ± 0.10 e	0.14 ± 0.00 d
ZXWC-M1	5.05 ± 0.03 a	94.79 ± 0.45 e	2.72 ± 0.01 d	0.85 ± 0.02 c	29.14 ± 0.31 b	0.33 ± 0.00 d	0.69 ± 0.02 f	0.32 ± 0.00 a
ZXWC-M2	4.85 ± 0.03 b	199.04 ± 0.82 a	4.81 ± 0.01 a	0.92 ± 0.02 b	22.52 ± 0.18 d	0.52 ± 0.00 a	2.40 ± 0.08 a	0.22 ± 0.00 c
ZXWC-M3	5.06 ± 0.05 a	131.11 ± 0.85 b	3.68 ± 0.00 b	0.86 ± 0.02 c	29.17 ± 0.42 b	0.15 ± 0.00 f	1.28 ± 0.11 d	0.21 ± 0.00 c
ZXWC-M4	5.09 ± 0.01 a	101.00 ± 1.08 c	2.87 ± 0.01 c	1.03 ± 0.01 a	29.92 ± 0.27 a	0.35 ± 0.00 c	2.02 ± 0.07 c	0.24 ± 0.00 b
ZXWC-M5	4.87 ± 0.01 b	98.69 ± 0.28 d	2.52 ± 0.01 f	0.85 ± 0.03 c	30.48 ± 0.32 a	0.36 ± 0.01 b	2.23 ± 0.12 b	0.22 ± 0.00 c

Different lowercase letters within the same column indicate significant differences according to Duncan’s test. (*p* < 0.05). XCB-M1~M5: Soil samples from co-cultivation with five *A. mellea* of different sources in XCB; XCB-CK: Soil samples in XCB with no GE–*A. mellea* co-cultivation; ZXWC-M1~M5: Soil samples from co-cultivation with five *A. mellea* of different sources in ZXWC; ZXWC-CK: Soil samples in ZXWC with no GE–*A. mellea* co-cultivation. TOC: total organic carbon; TN: total nitrogen; TP: total phosphorus; TK: total potassium; AN: alkali-hydrolyzable nitrogen; AP: available phosphorus; AK: available potassium.

**Table 3:** Bacterial abundance and diversity in the rhizosphere soil of GE ($\bar{x}$ ± s, *n* = 3).

Experimental Variants	Shannon	Simpson	Chao1	Ace
XCB-M1	9.47 ± 0.07 ab	0.99 ± 0.00 a	7386.14 ± 33.99 a	7964.02 ± 59.60 a
XCB-M2	9.76 ± 0.06 a	0.99 ± 0.00 a	7760.93 ± 176.05 a	8370.44 ± 196.83 a
XCB-M3	9.82 ± 0.03 a	0.99 ± 0.00 a	7876.11 ± 533.28 a	8476.87 ± 555.31 a
XCB-M4	9.68 ± 0.12 a	0.99 ± 0.00 a	8021.11 ± 71.15 a	8633.52 ± 86.53 a
XCB-M5	9.84 ± 0.32 a	0.99 ± 0.00 a	8151.22 ± 817.20 a	8780.38 ± 811.26 a
XCB-CK	9.31 ± 0.30 b	0.98 ± 0.01 b	7360.18 ± 295.85 a	7942.03 ± 327.56 a
ZXWC-M1	10.03 ± 0.27 a	0.99 ± 0.00 a	8282.91 ± 697.86 a	8911.52 ± 718.32 a
ZXWC-M2	10.20 ± 0.04 a	0.99 ± 0.00 a	7815.01 ± 64.41 a	8404.57 ± 61.08 a
ZXWC-M3	10.17 ± 0.13 a	0.99 ± 0.00 a	8008.99 ± 818.92 a	8636.46 ± 848.12 a
ZXWC-M4	10.06 ± 0.17 a	0.99 ± 0.00 a	7861.78 ± 291.11 a	8437.33 ± 294.33 a
ZXWC-M5	10.15 ± 0.05 a	0.99 ± 0.00 a	7619.17 ± 121.47 a	8234.71 ± 159.11 a
ZXWC-CK	10.08 ± 0.28 a	0.99 ± 0.00 a	7776.25 ± 948.89 a	8398.53 ± 987.91 a

Different lowercase letters within the same column indicate significant differences according to Duncan’s test. (*p* < 0.05). XCB-M1~M5: Soil samples from co-cultivation with five *A. mellea* of different sources in XCB; XCB-CK: Soil samples in XCB with no GE–*A. mellea* co-cultivation; ZXWC-M1~M5: Soil samples from co-cultivation with five *A. mellea* of different sources in ZXWC; ZXWC-CK: Soil samples in ZXWC with no GE–*A. mellea* co-cultivation.

**Table 4:** Fungal abundance and diversity in the rhizosphere soil of GE ($\bar{x}$ ± s, *n* = 3).

Experimental Variants	Shannon	Simpson	Chao1	Ace
XCB-M1	6.59 ± 0.17 b	0.96 ± 0.01 a	2182.82 ± 59.00 b	2208.83 ± 52.34 b
XCB-M2	6.73 ± 0.14 b	0.97 ± 0.01 a	2349.63 ± 60.05 a	2389.86 ± 46.23 a
XCB-M3	6.68 ± 0.05 b	0.97 ± 0.00 a	2292.19 ± 97.35 ab	2332.57 ± 90.35 ab
XCB-M4	6.73 ± 0.11 b	0.97 ± 0.00 a	2273.36 ± 15.88 ab	2318.19 ± 19.74 ab
XCB-M5	6.71 ± 0.16 b	0.96 ± 0.01 a	2279.25 ± 101.48 ab	2307.94 ± 108.12 ab
XCB-CK	6.97 ± 0.09 a	0.97 ± 0.00 a	2184.60 ± 74.64 b	2200.75 ± 68.30 b
ZXWC-M1	6.49 ± 0.25 bc	0.93 ± 0.01 b	2476.84 ± 70.71 a	2526.65 ± 66.36 a
ZXWC-M2	6.95 ± 0.05 b	0.95 ± 0.01 ab	2557.06 ± 49.95 a	2583.16 ± 53.96 a
ZXWC-M3	6.72 ± 0.37 bc	0.93 ± 0.03 b	2404.47 ± 121.51 a	2442.05 ± 118.53 a
ZXWC-M4	6.34 ± 0.17 c	0.92 ± 0.02 b	2419.64 ± 57.00 a	2450.04 ± 35.89 a
ZXWC-M5	7.03 ± 0.42 ab	0.96 ± 0.02 ab	2519.81 ± 139.20 a	2514.68 ± 129.79 a
ZXWC-CK	7.56 ± 0.43 a	0.98 ± 0.01 a	2571.94 ± 106.49 a	2594.53 ± 66.94 a

Different lowercase letters within the same column indicate significant differences according to Duncan’s test. (n = 3, *p* < 0.05). XCB-M1~M5: Soil samples from co-cultivation with five *A. mellea* of different sources in XCB; XCB-CK: Soil samples in XCB with no GE–*A. mellea* co-cultivation; ZXWC-M1~M5: Soil samples from co-cultivation with five *A. mellea* of different sources in ZXWC; ZXWC-CK: Soil samples in ZXWC with no GE–*A. mellea* co-cultivation.

**Table 5:** Natural conditions of the experimental sites.

Experimental Plot Code	Experimental Fields	Altitude	Latitude	Longitude
XCB	Yunnan Senhao Mushroom Industry Co., LTD., Xiaocaoba, Yiliang, Zhaotong, China	1882.5 m	27°47′17″ N	104°18′22″ S
ZXWC	Xuancheng *Gastrodia elata* Planting Professional Cooperative, Wanchang Town, Zhenxiong, Zhaotong, China	1624.3 m	27°63′89″ N	104°50′78″ S

**Table 6:** Relevant information on *Armillaria* from different sources.

*A. mellea* Strain Code	Origin	Cultivation Substrate	Specification (g/Bottle)	Sequence ID
M1	Shanxi Sensheng Fungus Industry Technology Co., Ltd., Xi’an, Shanxi, China	Branch	645.5	MT673937.1
M2	Hubei Hongsheng Fungus Industry Co., Ltd., Suizhou, Hubei, China	Maize kernel	808.5	KF156775.1
M3	Yongqian Strain Factory, Tuohe Village, Zhaotong, China	Branch	655.0	MZ851983.2
M4	Zhaotong Gastrodia Elata Research Institute, Zhaotong, China	Branch	1087.5	MT647067.1
M5	Yunnan Senhao Fungus Industry Co., Ltd., Zhaotong, China	Cottonseed hull	1047.0	MZ851983.1

**Table 7:** Gradient elution order.

Time/Min	A (0.05% Phosphoric Acid)	B (100% Acetonitrile)
0~8	98	2
8~21	98~92	2~8
21~30	92~88	8~12
30~50	88~76	12~24

**Table 8:** Calibration parameters and recovery results for eight target compounds (n = 3).

Component	Regression Equation	Linear Range (mg·mL^−1^)	R^2^	Average Recovery (%)	RSD (%)
GAS	y_1_ = 1499.05 x_1_ + 2924.37	0.156–0.779	0.9999	99.65	1.64
HBA	y_2_ = 5183.80 x_2_ + 14,848.4	0.011–0.066	0.9997	103.36	2.36
PHBA	y_3_ = 54,723.3 x_3_ − 11,536.6	0.007–0.033	0.9993	100.94	2.35
4HBD	y_4_ = 66,247.9 x_4_ + −1082.76	0.016–0.081	0.9998	104.56	2.13
PE	y_5_ = 759.088 x_5_ + 3313.31	0.138–0.695	0.9999	98.34	1.58
PB	y_6_ = 1044.90 x_6_ + 2570.46	0.052–0.268	0.9999	111.78	2.27
PC	y_7_ = 803.118 x_7_ − 1087.01	0.093–0.458	0.9999	101.15	1.71
PA	y_8_ = 1077.65 x_8_ + 15 840.0	0.005–0.084	0.9999	101.41	1.27

GAS, gastrodin; HBA, p-hydroxybenzyl alcohol; PHBA, p-hydroxybenzoic acid; HBD, p-hydroxybenzaldehyde; PE, parishin E; PB, parishin B; PC, parishin C; PA, parishin A. In the regression equations (y = ax + b), y represents the peak area, and x represents the compound concentration (mg·mL^−1^). Recovery experiments were performed in triplicate (n = 3).

## Data Availability

The authors confirm that the data supporting the findings of this study are available within the article.

## Supplementary Materials

The following supporting information can be downloaded at: https://www.mdpi.com/article/10.3390/plants15091329/s1, Table S1: Classification standards for soil pH in arable land; Table S2: Statistics of sequencing data information for soil samples; Table S3: Relative Abundances of Fungal KEGG Metabolic Pathways in the Rhizosphere Soil.

## Abbreviations

The following abbreviations are used in this manuscript:

XCB	Xiaocaoba Town, Yiliang County, Zhaotong City
ZXWC	Wanchang Town, Zhenxiong County, Zhaotong City
GAS	Gastrodin
HBA	*p*-Hydroxybenzyl alcohol
PHBA	*p*-Hydroxybenzoic acid
HBD	4-Hydroxybenzaldehyde
PE	Parishin E
PB	Parishin B
PC	Parishin C
PA	Parishin A
TOC	Soil Total Organic Carbon
TN	Soil Available Nitrogen
TP	Soil Total Phosphorus
TK	Soil Total Potassium
AN	Soil Available Nitrogen
AP	Soil Available Phosphorus
AK	Soil Available Potassium
