# Supplementary material for: Effects of Different Sources of Armillaria mellea Co-Cultivation on the Quality and Soil Microecology of Gastrodia elata
Source: Plants (Basel). 2026 Apr 27;15(9):1329. doi: 10.3390/plants15091329 (PMC13165016; doi:10.3390/plants15091329)
Supplement: Supplementary file 1 [file plants-15-01329-s001.zip › table.pdf]

Supplementary Materials

Table S1. Classification standards for soil pH in arable land.

Level	Strongly Acidic	Acidic	Slightly Acidic	Neutral	Slightly Alkaline
Standard	＜4.5	4.5~5.5	5.5~6.5	6.5~7.5	7.5~8.5

Table S2. Statistics of Sequencing Data Information for Soil Samples.

Sample Name	16S			ITS		
	Total Sequences/Read	High - Quality Sequences/Read	Quality Control Efficiency/%	Total Sequences/Read	High - Quality Sequences/Read	Quality Control Efficiency/%
XCB-M1-a	115248	98314	85.31	97405	93396	95.88
XCB-M1-b	110855	95335	86	96164	91284	94.93
XCB-M1-c	104705	90063	86.02	104577	99291	94.95
XCB-M2-a	108196	91446	84.52	107653	102742	95.44
XCB-M2-b	111125	95937	86.33	104167	99133	95.17
XCB-M2-c	114635	98037	85.52	90793	86504	95.28
XCB-M3-a	123374	105274	85.33	93085	89095	95.71
XCB-M3-b	94089	81632	86.76	108715	104125	95.78
XCB-M3-c	116132	100548	86.58	102008	96870	94.96
XCB-M4-a	120879	103125	85.31	102920	97961	95.18
XCB-M4-b	114836	99766	86.88	96584	92000	95.25
XCB-M4-c	111518	94910	85.11	91565	87302	95.34
XCB-M5-a	104308	90853	87.1	111195	106054	95.38
XCB-M5-b	117045	100474	85.84	103360	97822	94.64
XCB-M5-c	128637	110125	85.61	121993	115512	94.69
XCB-CK-a	108385	94268	86.98	99616	95235	95.6
XCB-CK-b	112893	97982	86.79	92647	88184	95.18
XCB-CK-c	114732	99261	86.52	93074	88900	95.52
ZXWC-M1-a	128318	109127	85.04	94605	90657	95.83
ZXWC-M1-b	109530	94347	86.14	95043	90938	95.68
ZXWC-M1-c	110053	94310	85.7	98766	93935	95.11
ZXWC-M2-a	102426	88666	86.57	97847	93237	95.29
ZXWC-M2-b	108769	93356	85.83	108937	103621	95.12
ZXWC-M2-c	100586	86186	85.68	94304	89627	95.04
ZXWC-M3-a	85634	74623	87.14	79587	76164	95.7
ZXWC-M3-b	108302	93657	86.48	83818	80109	95.57
ZXWC-M3-c	117834	101404	86.06	87820	83611	95.21
ZXWC-M4-a	111102	96214	86.6	80302	76602	95.39
ZXWC-M4-b	102306	89054	87.05	85042	81073	95.33
ZXWC-M4-c	100278	86639	86.4	82983	79349	95.62
ZXWC-M5-a	98703	85834	86.96	90942	87052	95.72
ZXWC-M5-b	100471	87069	86.66	92712	88541	95.5
ZXWC-M5-c	104003	90475	86.99	89459	85229	95.27

Sample Name	16S			ITS		
	Total Sequences/Read	High - Quality Sequences/Read	Quality Control Efficiency/%	Total Sequences/Read	High - Quality Sequences/Read	Quality Control Efficiency/%
ZXWC-CK-a	84370	74377	88.16	100926	96484	95.6
ZXWC-CK-b	121852	103672	85.08	105641	100234	94.88
ZXWC-CK-c	113903	97387	85.5	101773	97804	96.1

XCB-M1~M5: Soil samples from co-cultivation with five *A. mellea* of different sources in XCB, XCB-CK: Soil samples in XCB with no GE–*A. mellea* co-cultivation; ZXWC-M1~M5: Soil samples from co-cultivation with five *A. mellea* of different sources in ZXWC, ZXWC-CK: Soil samples in ZXWC with no GE–*A. mellea* co-cultivation.

Table S3. Relative Abundances of Fungal KEGG Metabolic Pathways in the Rhizosphere Soil of GE (x¯±s, n=3) (x̄±s, n=3).

KEGG Metabolic Pathways	Relative Abundances /%											
	XCB						ZXWC					
	CK	M1	M2	M3	M4	M5	CK	M1	M2	M3	M4	M5
Carbohydrate Metabolism	12.76	12.74	12.73	12.71	12.87	12.89	12.80	12.73	12.49	12.47	12.70	12.63
Amino Acid Metabolism	12.31	12.20	12.20	12.20	12.10	12.07	12.05	12.09	12.1%	12.20	12.10	12.13
Energy Metabolism	7.11	6.99	7.01	7.02	6.97	6.97	7.07	7.06	7.22	7.19	7.02	7.22
Metabolism of Cofactors and Vitamins	6.87	6.82	6.81	6.82	6.79	6.81	6.78	6.80	6.77	6.80	6.78	6.79
Nucleotide Metabolism	5.07	5.14	5.14	5.14	5.19	5.21	5.19	5.16	5.09	5.10	5.16	5.11
Xenobiotics Biodegradation and Metabolism	4.53	4.40	4.39	4.40	4.31	4.27	4.28	4.34	4.47	4.45	4.34	4.43
Lipid Metabolism	3.53	3.56	3.55	3.55	3.57	3.56	3.52	3.53	3.49	3.50	3.55	3.48
Metabolism of Other Amino Acids	2.82	2.74	2.73	2.74	2.69	2.69	2.68	2.69	2.72	2.73	2.67	2.73
Metabolism of Terpenoids and Polyketides	2.49	2.56	2.56	2.55	2.63	2.61	2.55	2.56	2.46	2.45	2.59	2.46
Glycan Biosynthesis and Metabolism	2.39	2.35	2.35	2.36	2.37	2.40	2.38	2.35	2.29	2.30	2.33	2.34

Biosynthesis of Other Secondary Metabolites	1.04	0.98	0.97	0.97	0.97	0.96	0.94	0.95	0.93	0.93	0.93	0.95
