# Supplementary figures and images for: Effects of Different Sources of Armillaria mellea Co-Cultivation on the Quality and Soil Microecology of Gastrodia elata
Source: Plants (Basel). 2026 Apr 27;15(9):1329. doi: 10.3390/plants15091329 (PMC13165016; doi:10.3390/plants15091329)

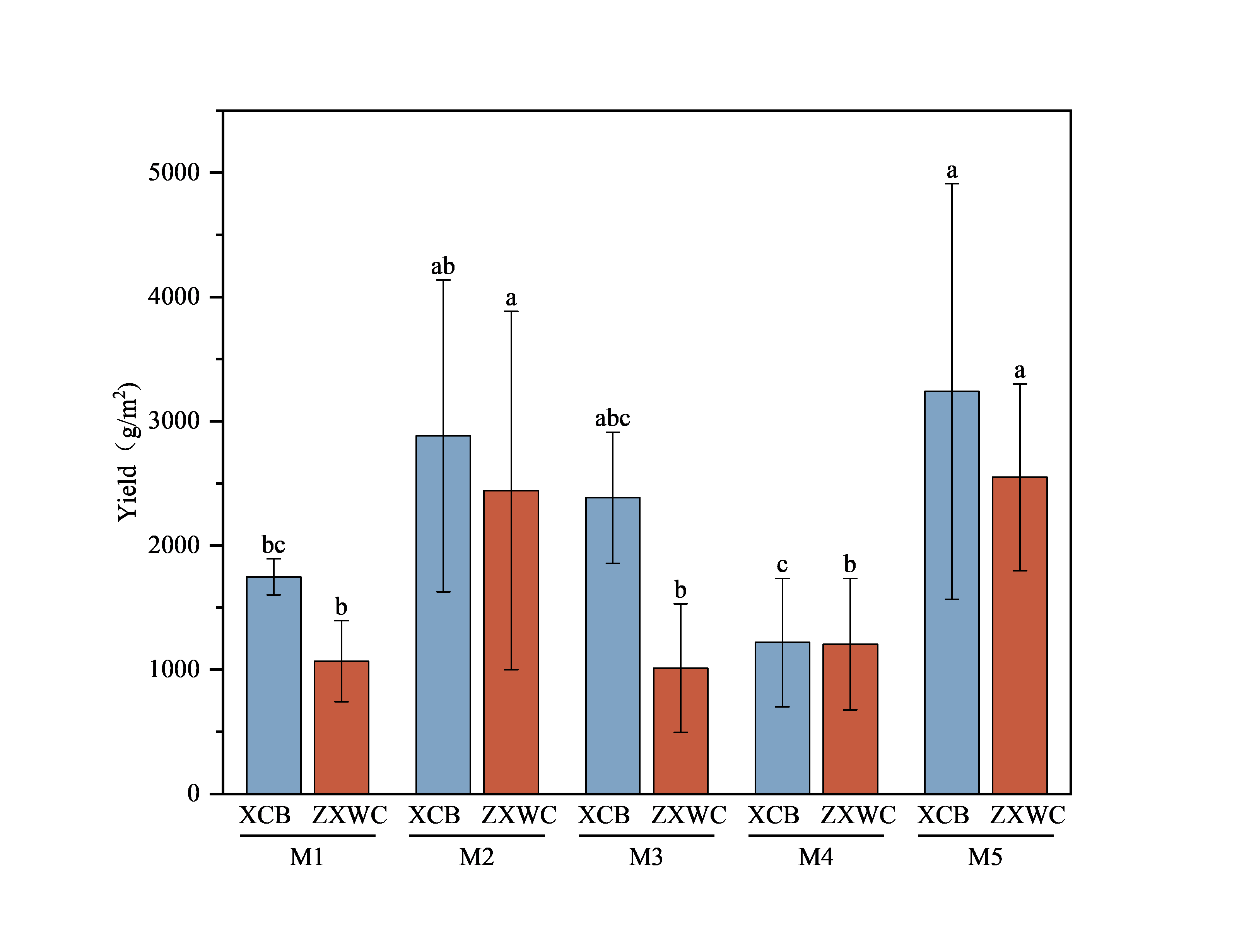

Supplement: Supplementary file 1 [file plants-15-01329-s001.zip › Figure/Figure 1(a).png]

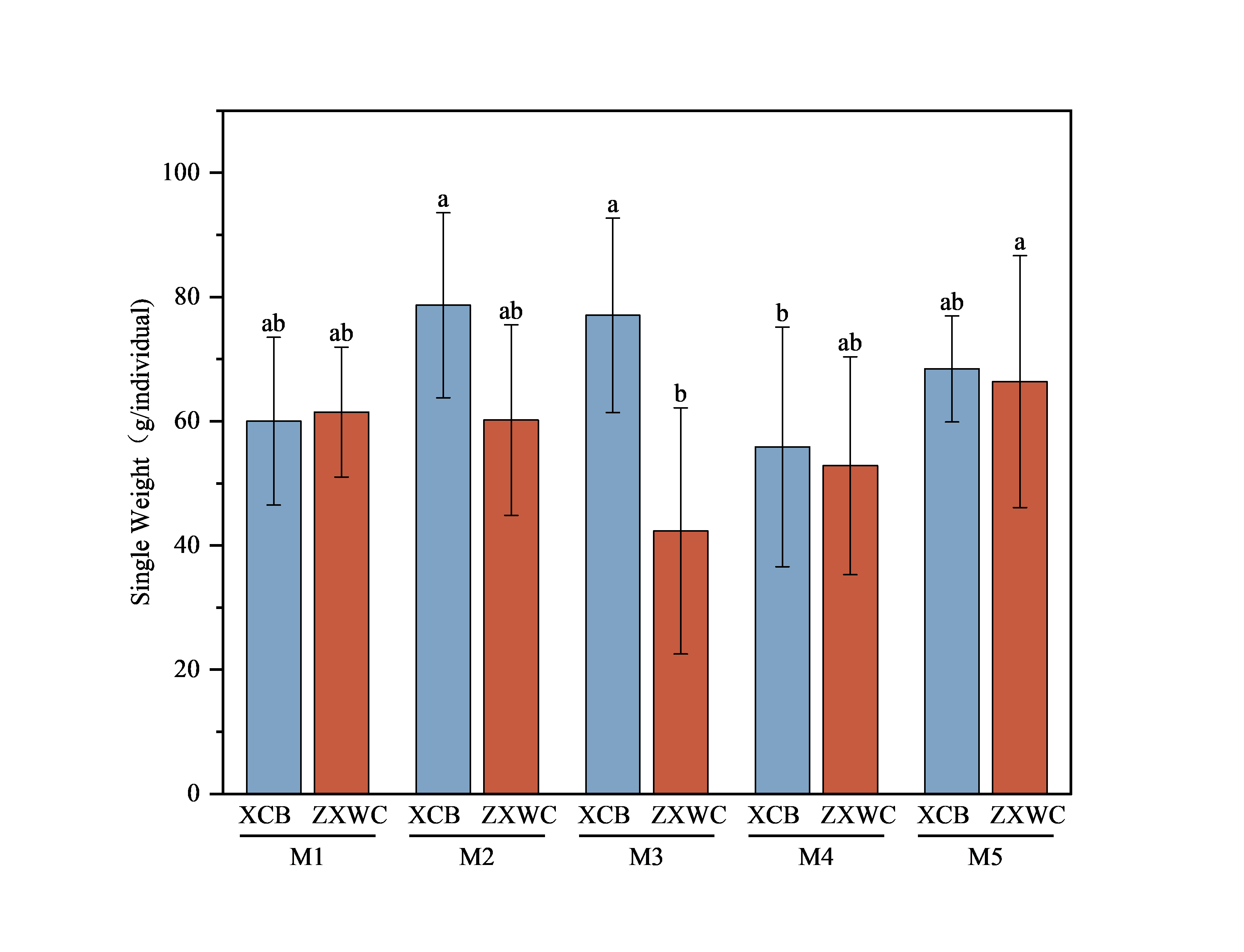

Supplement: Supplementary file 1 [file plants-15-01329-s001.zip › Figure/Figure 1(b).png]

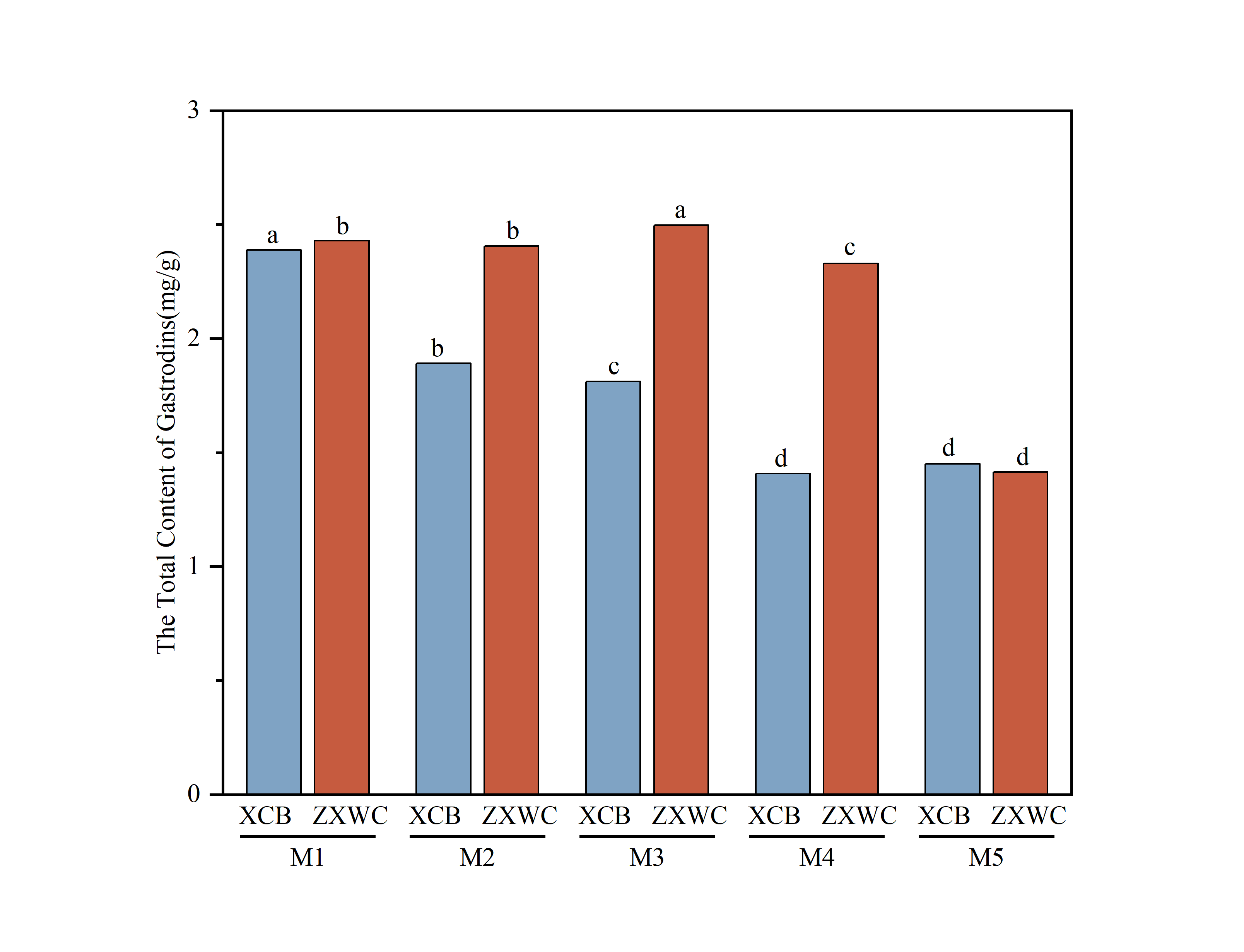

Supplement: Supplementary file 1 [file plants-15-01329-s001.zip › Figure/Figure 2(a).png]

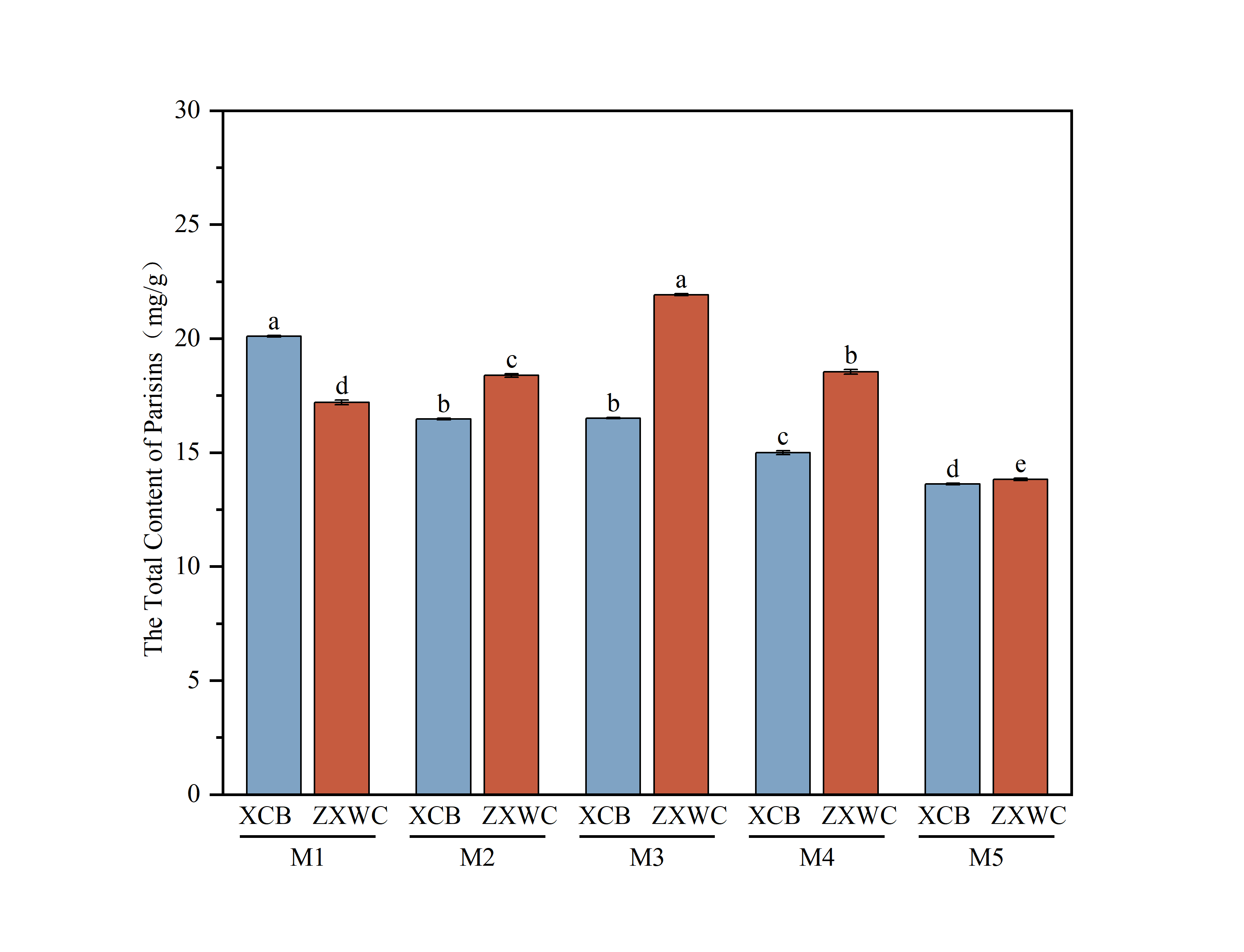

Supplement: Supplementary file 1 [file plants-15-01329-s001.zip › Figure/Figure 2(b).png]

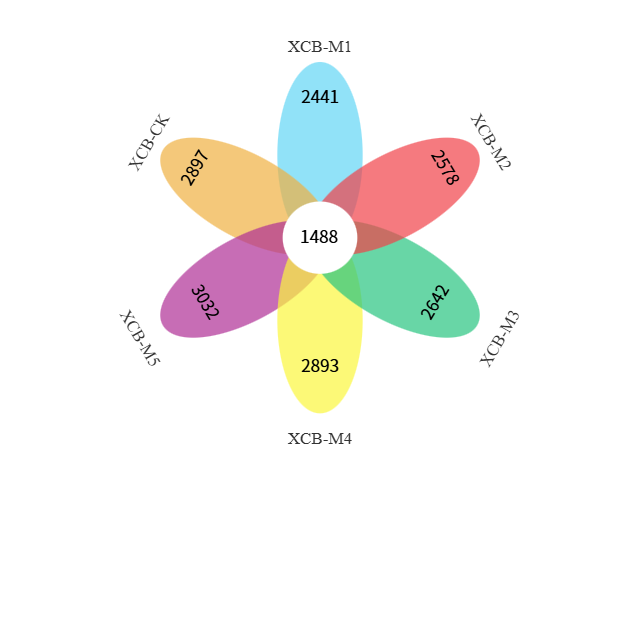

Supplement: Supplementary file 1 [file plants-15-01329-s001.zip › Figure/Figure 3(a).png]

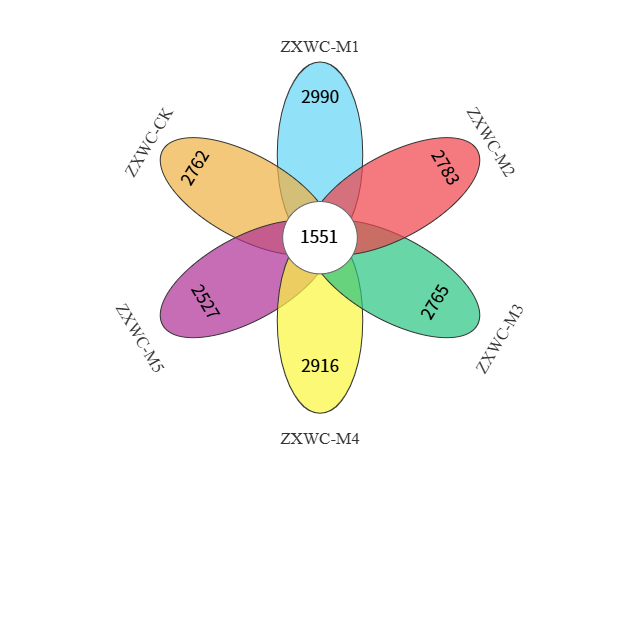

Supplement: Supplementary file 1 [file plants-15-01329-s001.zip › Figure/Figure 3(b).png]

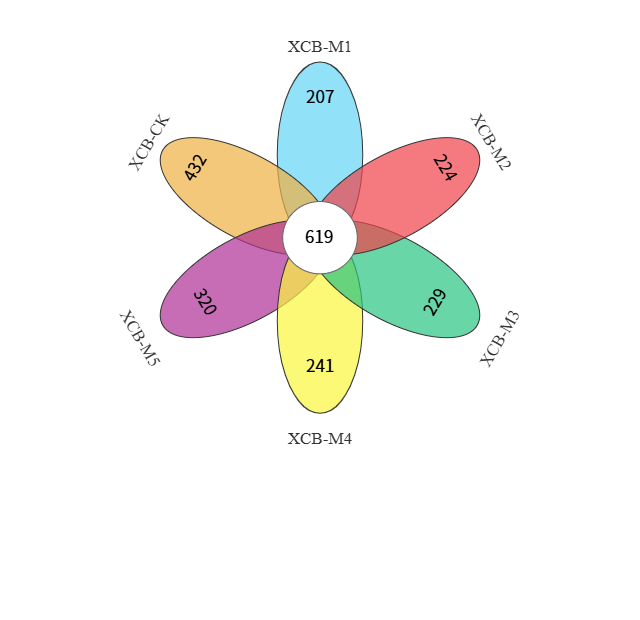

Supplement: Supplementary file 1 [file plants-15-01329-s001.zip › Figure/Figure 4(a).png]

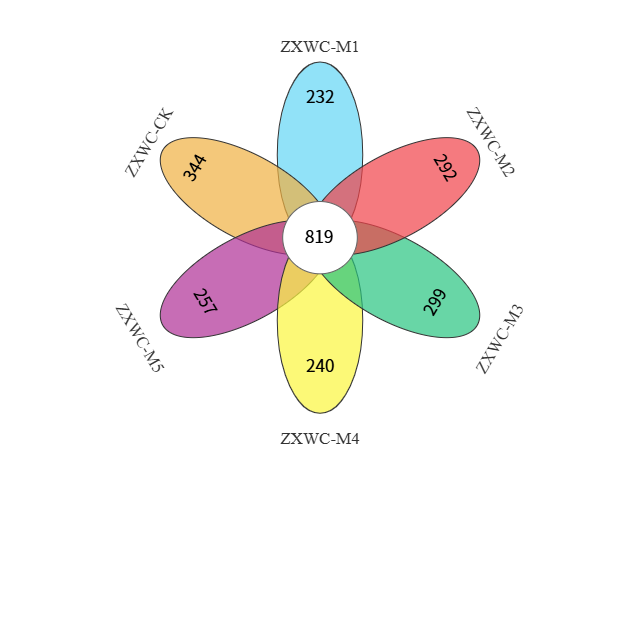

Supplement: Supplementary file 1 [file plants-15-01329-s001.zip › Figure/Figure 4(b).png]

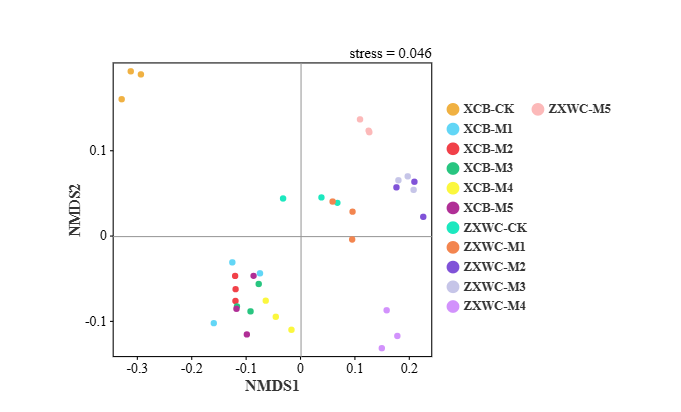

Supplement: Supplementary file 1 [file plants-15-01329-s001.zip › Figure/Figure 5(a).png]

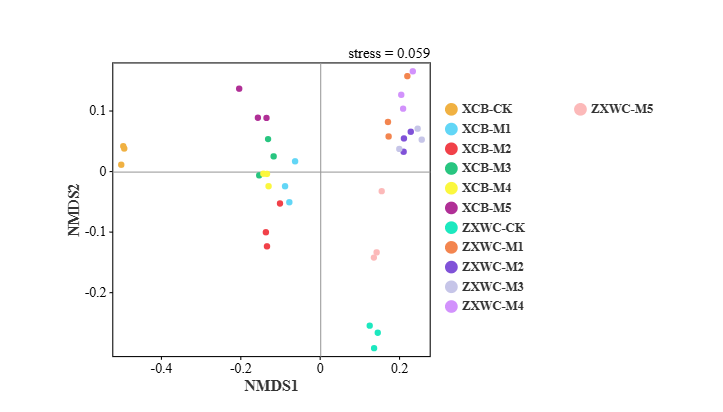

Supplement: Supplementary file 1 [file plants-15-01329-s001.zip › Figure/Figure 5(b).png]

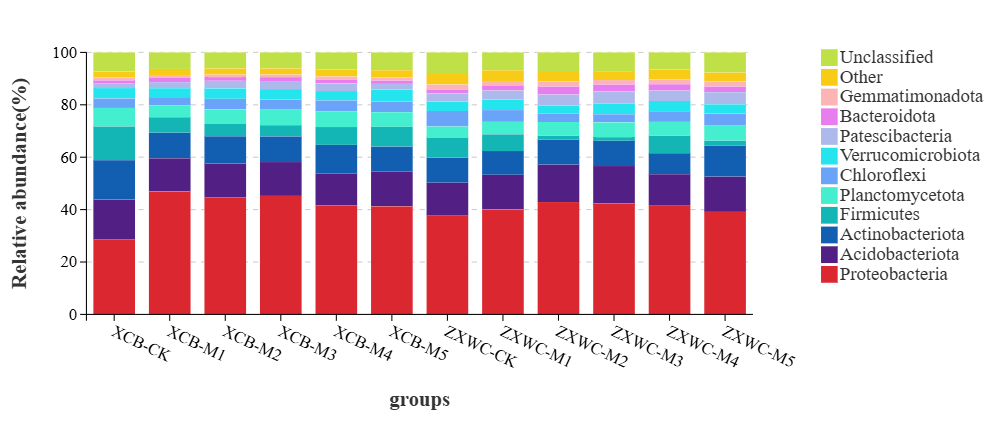

Supplement: Supplementary file 1 [file plants-15-01329-s001.zip › Figure/Figure 6(a).png]

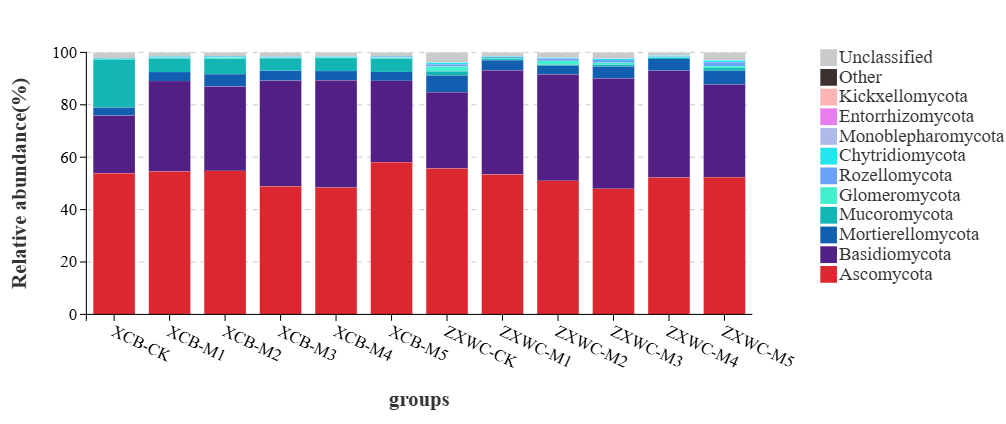

Supplement: Supplementary file 1 [file plants-15-01329-s001.zip › Figure/Figure 6(b).png]

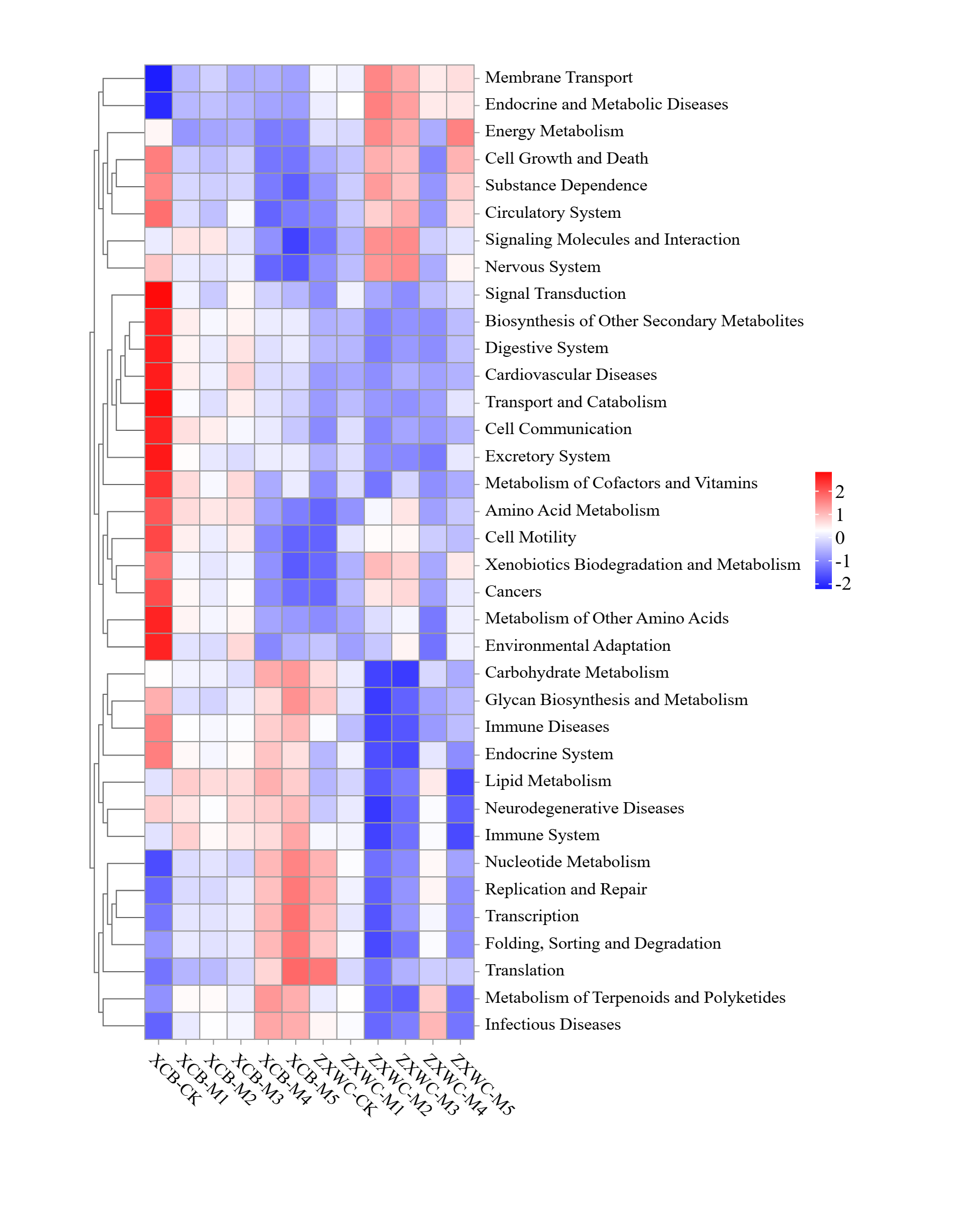

Supplement: Supplementary file 1 [file plants-15-01329-s001.zip › Figure/Figure 7(a).png]

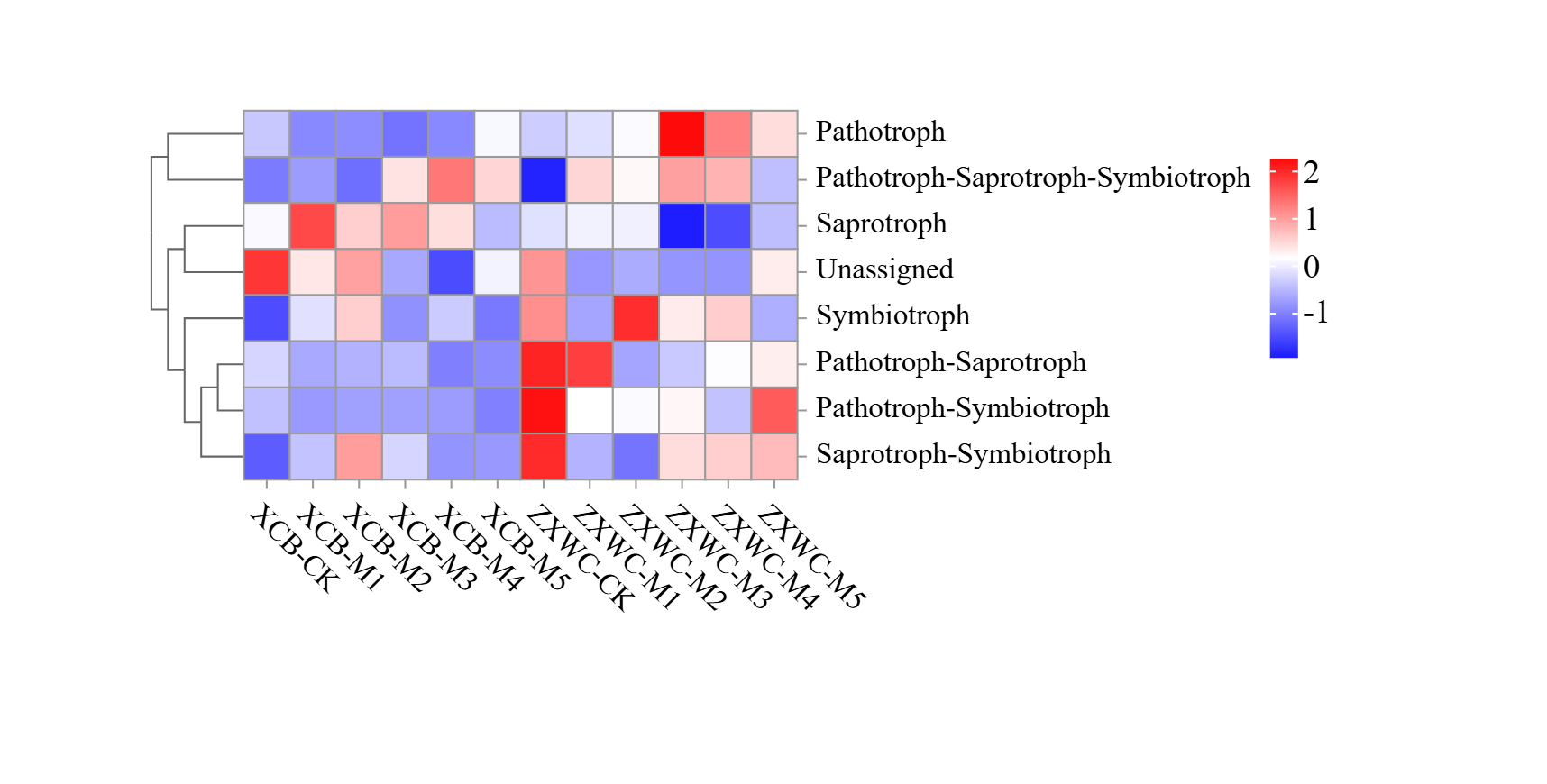

Supplement: Supplementary file 1 [file plants-15-01329-s001.zip › Figure/Figure 7(b).png]

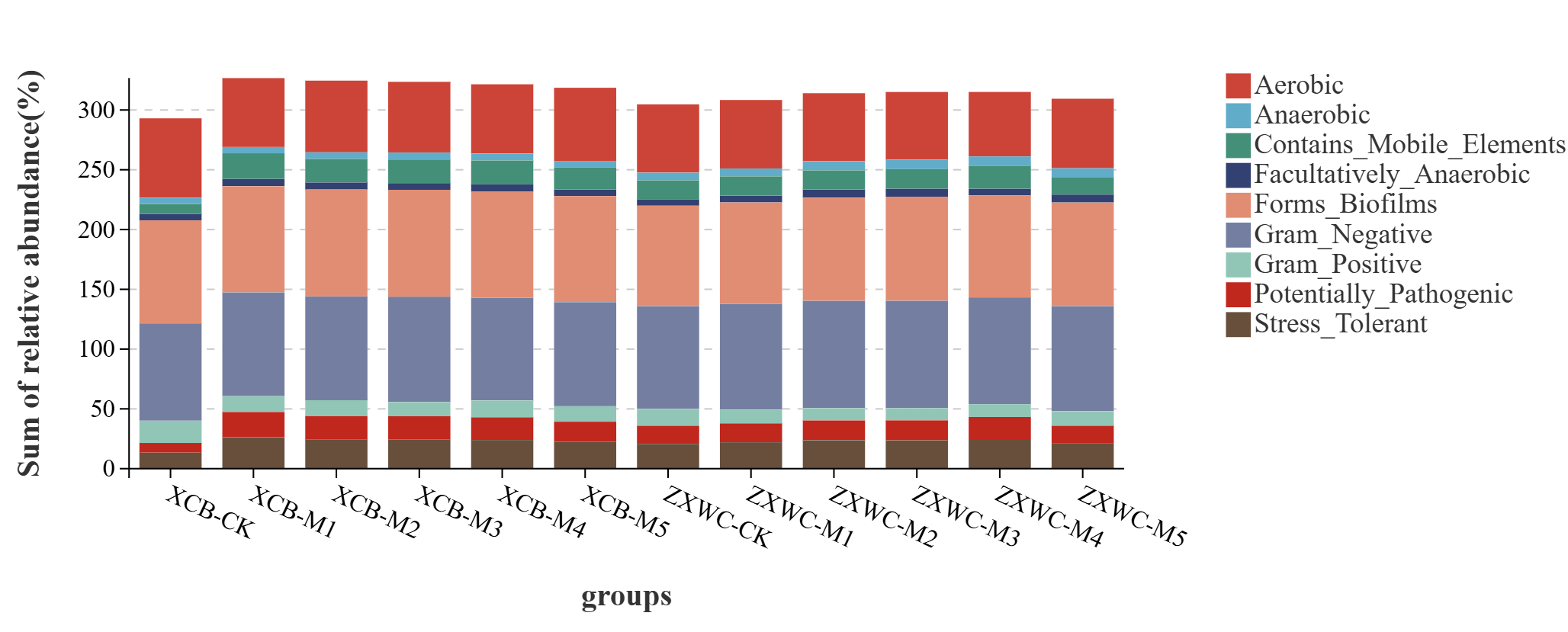

Supplement: Supplementary file 1 [file plants-15-01329-s001.zip › Figure/Figure 7(c).png]
